# Chapter I: Mind and Disposition: Abandoned—Fire

**Published:** 1889

**Authors:** Edmund J. Lee


### CHAPTER I.

### MIND AND DISPOSITION.

**Abandoned**, feels as if: carb-an. cyc. hura. k-ca. lam. stram.
— — she thinks she is left alone in the world, etc.: plat.
— — See Forsaken.
**Absent**-minded, see Mind, absence of, and compare with Forgetful.
**Absorbed** in self: aloe. caus. chin. *cocc.* cyc. ign. mur ac. nat-c. ol-an. phel. rheum. sars. stann.
— — — after eating: aloe.
— — — in morning: nat-c.
— — — during menses: mur-ac.
— (buried) in thought: Acon. am-m. bell. bov. cann-i. canth. cham. chin. *cocc.* con. cyc. grat. ham. merc. mosch. nit-ac. phel. phos. spig.
— — — alternating with frivolity: arg-n.
— — — evening: am-m.
— — — as to what would become of him: nat-m.
— compare with Thoughtful, etc.
**Abusive**: alcoh. am-c. anac. bell. borax. caus. coral. dulc. gal-ac. hydrph. hyos. ipec. lyc. mosch. nit-ac. *nx-v.* petr. plb. stram. verat. viol-tr.
— without being angry: Dulc.
— with the pains: coral.
— evening: am-m.
— **inclined** to be: atrop. caus. *con.* elæ. ran-b. *sep.*
**Activity**, desires: agar. ars. *bar.* bry. clem. coca. cocc. hyos. hyper. ign. iris. lach. led. lil-t. mezer. mosch. mur-ac. nat-s. *op.* phos. rhus. *sep.* stann. verat.
— mental and bodily; cannot do things fast enough: *aur.*
— alternating with exhaustion: *aloe.*
— in business: brom. manc.
— mental: see under Mind.
— with physical debility: mosch.
**Acutness** (increased of mind): *anac.* asaf. *aur. coff.* lach. *op.* verat. viol-od.

**Affectionate** [fond, loving disposition]: acon. anac. borax. carb-an. carb-v. hura. ign. nx-v. ox-ac. par. phos. plat. seneg.
— love for every one about him, during and after fainting: hura.
**Affronts** or offenses, ailments from. See name of the special cause.
**Ambition**, loss of: apoc. dios. erig. nat-p. petr. *sep.*
— — — in damp, cloudy weather: sang.
**Amorous**: acon. ant-c. bell. calad. calc. cann-s. *canth.* carb-v. chin. coff. coloc. con. croc. graph. *hyos.* ign. k-ca. lach. lil-t. lyc. men. merc. mosch. nat-c. nat m. nx-m. nx-v. op. orig. *phos. plat.* plb. puls. rhus. ruta. sabin. selen. sep. sil. staph. *stram.* sulph. thu. verat. verb. znc.
— fits: acon. ant-c. hyos. op. stram. verat.
**Amusement**, averse to: men.
— desire for: lach.
**Anger** (bad temper, irascibility, etc.): *acon.* aloe. ambr. am-c. *anac.* ang. apis. arg-n. arn. ars. asar. *aur.* bar. *bell. bry.* bufo. calad. calc. calc-p. cann-s. canth. caps. carb-an. *carb-v.* caus. *cham.* chel. chin. cimic. cinnb. clem. cocc. coff. coloc. con. cop. *croc.* cyc. dig. dros. eupion. ferr. fl-ac. gran. graph. ham. hell. hyos. hydr. ign. iod. ipec. k-ca. lach. led. *lyc.* mag-s. mang. meph. merc. merl. mezer. mur-ac. nat-c. *nat-m.* nicc. nit-ac. *nx-v.* olnd. op. osm. *petr. phos.* plat. psor. puls. ran-b. ratan. ruta. sabad. sang. seneg. sep. sil. spig. squil. stann. *staph.* stront. *sulph.* sul-ac. tarent. thu. trill. verat. znc.
— See also Ill-humor, Rage, etc.
— **ailments**, from or after anger, vexation, etc.: ACON. alum. ant-t. *ars.* aur. *bell. bry.* calc. CHAM. chin. cimic.

cocc. *coff.* COLOC. croc. cupr. hyos. IGN. ipec. *lyc.* mag-c. mag-m. manc. mezer. nat-c. nat-m. nx-m. NX-V. *op.* petr. *phos.* ph-ac. PLAT. *puls.* ran-b. rhus. samb. secale. selen. sep. sil. stann. *staph.* stram. sulph. verat. znc.
— anger, with anxiety: ACON. alum. ARS. aur. *bell.* bry. calc. *cham.* cocc. coff. *cupr.* hyos. *ign.* lyc. nat-c. nat-m. NX-V. *op.* petr. phos. *plat. puls.* rhus. samb. sep. stann. stram. sulph. verat.
— anger, with fright: ACON. *bell.* calc. cocc. cupr. IGN. nat-c. *nx-v. op.* petr. *phos. plat. puls.* samb. sep. sulph. znc.
— anger, with silent grief and sorrow: alum. ars. aur. bell. *cocc. coloc.* hyos. IGN. *lyc.* nat-c. nat-m. nx-v. phos. *ph-ac.* plat. puls. *staph.* verat.
— anger, with indignation: COLOC. ipec. *nx-v.* plat. *staph.*
— anger, with vehemence, passion: ACON. aur. bell. *bry.* CHAM. coff. hyos. lyc. nat-m. NX-V. phos. sep. sulph. verat. znc.
— **alternating** with cheerfulness: aur. caps. croc. ign. stram.
— — — quick repentance: croc. mezer. vinc-m. sulph.
— **causeless**: chel. mezer.
— **coition**, after: *calc.*
— **consoled**, when: *nat-m.*
— **contradiction**, from: *aur. bry.* ferr. ign. *nicc.* nx-v. op. petr. *sil.*
— **diarrhœa**, with: gnap.
— **drinking** coffee or wine, while: chlor.
— **evening**, in: am-c. bry. *croc.* nicc. op. petr.
— **forgetfulness**, during: hydr.
— **fever**, during: hipp.
— **followed** by: headache: mezer. petr. plat.
— — — red face and chilliness: bry.
— — — red tip of nose: vinc-m.
— — — chilliness, heat and vomiting: nx-v.
— — — rage, violence and heat: cham.
— — — throwing away things or pushing away table: staph.
— with **indignation**: coloc. *staph.*
— **morning**, in: nx-v. petr. sep. *sulph.*
— — on waking: k-ca.
— **news**, at unpleasant: calc-p.
— **noise**, at: ipec.
— **past** events, over: sep.
— **reproaches**, at: croc.
— **thinking** of absent persons, on: aur.
— — of former vexations: calc.
— — of what may happen: sol-m.
— **trifles**, at: ars. bell. cann-s. cocc. croc. hell. ipec. lyc. meph. mezer. nat-c. nat-m. nit-ac. seneg. sep. squil. thu.
— **vehemence**, with: acon. ars. aur. bry. *cham.* grat. ign. lyc. nx-v. verat.
— — — — so that he could have stabbed any one: chin.
— — with suppressed: cham. *ign.* staph.

**Anguish** (agony): acet-ac. ACON. æth. aloe. alum. ambr. ant-t. arn. ARS. *aur. bell.* bov. calc. *cann-i.* carb-v. cedr. k-iod. murx. sep. tarent. trill. verat.
— causing restlessness: *ars.*
— during chill, heat, or sweat: *arn.*
— **eating**, while: sep.
— nausea, with: ailan. *ars.*
— **night**: ambr. arn.
— open air, amel: *cann-i.*
— palpitation, with: ars.
— during vomiting: ars. asar.

**Annoyance**, intolerant of any: ferr-p.
— see also Vexation.

**Answer**, does not: agar. alum. ambr. am-m. *arn.* atrop. bell. chin. cimic. cocc. *coloc.* euphr. hyos. mag-m. manc. merc. mosch. op. PH-AC. secale. *stann.* sul-ac. tabac. tarent. verat.
— — — at other times is loquacious: cimic.
— — — sings, talks, but will not answer questions: agar.
— **confusedly**, as though thinking of something else: mosch.
— **difficult**, is: chlol. *phos.* verat.
— **disconnected**, is: coff-t. *crotal.* phos. stram. strych.
— **hastily**: ars. bry. cimic. cocc. hepar. rhus. strych.
— **incoherently**. bell. cann-i. chlol. coff-t. hyos. phos.
— **incorrectly**: bell. hyos. merc. ph-ac.
— **irrelevantly**: cimic. hyos. nx-m. ph-ac. sul-ac. valer.
— **monosyllabic**: *ph-ac.*
— — "no" to all questions: crot-c.
— **repeats** the question first: znc.
— **slowly**: ars. cup-ac. hell. *merc. phos.* ph-ac. plb. secale. sul-ac. sulph. znc.
— **shortly**, abruptly, curtly: ars. cic. coff. jatr. *ph-ac.* plb sin-n. *stann.*
— **stupor** returns quickly after: arn. bapt. *hyos.*

— **unintelligibly**: coff-t. hyos. phos.
— **violently**, as if angry: rhus.
— see also under Delirium, Delusions, Silent, Speech, etc.

**Anthropophobia**: acon. aloe. alum. ambr. anac. aur. bar. bell. carb-an. chin. cic. con. cupr. hyos. k-bi. led. lyc. merc. *nat-c.* nat-m. phos. puls. rhus. selen. stann. sulph.

**Anxiety**: abrot. *acon.* acet-ac. *æth.* agar. agn. ailan. aloe. *alum. ambr.* am-c. am-m. *anac.* ang. ant-c. ant-t. *arg.* arg-n. *arn. ars.* asaf. *asar.* aur. *bar.* bar-m. *bell.* berb. bism. bov. *bry.* calad. *calc. camph.* cann-s. *canth.* caps. carb-an. *carb-v. caus. cham.* chel. *chin.* cic. cimic. cina. *cocc. coff. coloc. con.* croc. crotal. croton. cupr. cyc. *dig. dros.* euph. *evon. ferr.* fl-ac. *graph.* grat. *hell. hepar.* hyos. ign. iod. ipec. *jatr.* k-bro. k-ca. lach. lact. lam. *laur.* lil-t. *lyc.* mag-c. mag-m. *mag-s.* mang. marum. men. *merc.* mezer. *mosch. mur-ac. nat-c. nat m.* nicc. nitr. nit-ac. *nx-v.* olnd. op. petr. phel. *phos.* ph-ac. plat. *plb.* psor. *puls.* ran-b. ran-sc. raph. rheum. rhod. *rhus. ruta. sabad.* sang. *samb.* sars. *secale. seneg. sep.* sil. *spig. spong.* squil. *stann.* staph. *stram.* stront. sulph. sul-ac. *tabac.* thu. valer. *verat.* viol-od. viol-tr. znc.
— — and impeded breathing; compelling him to go out and be busy: laur.
— **abdominal**: am-m. bry. calen. carb-v. *cham. cupr.* euph. mosch. mur-ac. rhus. squil. sul-ac.
— **alternating** with indifference: nat-m.
— — with jollity: spig. spong.
— **apoplexy**, as if he would have: puls.
— — — — in morning: alum.
— **bad**, as if he had done something. See Conscience.
— **business**, about: anac.
— — as if engaged in a lawsuit: *nit-ac.*
— **cardiac** (in heart): ACON. ambr. anac. *ars.* AUR. BELL. brom. calc. camph. cann-s. canth. cast. caus. *cham.* cic. cocc. croc. cupr. cyc. evon. ferr. graph. hell. led. lyc. mag-m. men. merc. nit-ac. nx-v. olnd. op. phos. plat. *puls.* ran-b. rhus. secale. sep. sil. stram. sulph. verat. viol-tr.
— **causeless**: *bry.* phos. tabac.
— **children**, about his: acet-ac.
— **conscience**, of (as if guilty of a crime): *alum.* am-c. *ars.* **cact.** caus. chel. cina. *cocc.* cyc. dig. ferr. graph. *ign.* mag-s. *merc.* nat-m. nit-ac. *nx-v.* rheum. ruta. sil. stront. *sulph. verat.* znc.
— **death** (apprehensive of): *acon.* alum. am-c. *ars.* asaf. aur. caps. *cocc.* con. cupr. graph. hell. hepar. ipec. lyc. mosch. nat-m. nit-ac. *nx-v.* phos. ph-ac. phyt. plat. *puls. raph.* rheum. rhus. squil. sulph. verat.
— — morning: con.
— — — after waking: lyc.
— — evening: *phos.*
— — night: chel.
— — sitting bent over, while: rhus.
— — — aggravates: ph-ac.
— — toothache, during: olnd.
— — vertigo, during: nat-c.
— **dreaded** something bad. See Future.
— — — disagreeable: agar. *caus.* merc.
— — — serious, morning and afternoon: nx-v.
— **domestic** affairs, about: bar. puls. rhus. sep. sulph.
— **evil**, of: acon. agar. alum. am-c. anac. ant-c. arn. *ars.* aur. bar. bry. calad. carb-an. carb-v. caus. chin. cina. *cocc. coff.* cyc. dig. dros. dulc. euph. ferr. graph. hell. hyos. k-ca. lach. mag-c. men. merc. mur-ac. nat-m. nit-ac. nx-v. petr. phos. puls. rhus. ruta. sabin. sep. spig. spong. squil. staph. stront. sulph. sul-ac. thu. verat.
— — — morning on waking: mag-s. sulph.
— — — evening: graph.
— — — while walking in open air: cina.
— **fearful**, timid: *alum.* am-c. *am-m.* anac. ant-c. ant-t. ars. *aur.* bar. bry. calad. *calc. canth. caus.* chin. cic. cina. clem. cocc. *coff. dig.* dros. dulc. ferr. *graph.* hell. *hepar.* hyos. ign. k-ca. k-iod. lach. *mag c.* mang. men. *merc.* nat-c. nicc. nit-ac. nx-m. nx-v. phell. puls. rhus. ruta. sabin. samb. sep. *spig.* spong. staph. *stront.* sulph. tabac. thu. verat.
— **friends**, at home, about: phys.
— **future**, about: agar. alum. anac. ant-c. ant-t arn. bar. *bry.* calad. calc. calc-ac. caus. chel. chin. *cic.* con. cyc. *dig. dros.* dulc. euph. gins. hipp. lach.

mang. mur-ac. nat-c. *nat-m.* *nx-v.* petr. phos. ph-ac. psor. puls. ran-b. spig. stann. staph. sulph. thu. wies.
— **ghosts**, about, on waking from a dream: *sulph.*
— **health**, about: acon. am-c. arn. ars. bry. *calc.* ign. k-ca. lac-can. lach. nat-c. nit-ac. nx-m. nx-v. phos. ph-ac. psor. puls. sep. sil. staph. sulph.
— — about his disease: acet-ac. am-c. arg. arg-n. nit-ac. *phos.* sep.
— — fears consumption: sep.
— — especially during climacteric period: sil.
— **illness**, concerning: euph. nit ac. ph-ac. psor.
— — of others: cocc.
— — See also under Delusions, Fears, etc.
— **misfortune**, of. See Evil.
— **oppressive**: acon. alum. am-m. ant-t. *arn.* *ars.* *bell.* calad. calc. *canth.* *caus.* cham. chel. chin. cina. cocc. dig. euph. *graph.* iod. lact. men. *merc.* *mur-ac.* rhus. sep. sil. spig. stront. sulph. sul-ac. verat. znc.
— **pectoral** (in chest): acon. agar. am-c. ant-t. arn. ars. asaf. aur. bell. *bry.* calc. cann-s. caps. carb-an. chel. chin. cocc. colch. cop. ferr. guai. ign. iod. k-ca. lach. laur. lyc. merc. mezer. mosch. nat-c. nat m. nx-v. olnd. op. plb. prun. puls. rhus. samb. secale. seneg. sep. spig. spong. stann. staph. sulph. valer. verat.
— — anxiety in region of sternum, without pain, feels as if he must go out into open air and be busy: anac.
— — from chest to head: acon.
— **præcordial**: anac. bov. cann-s. chin. cic. con. dig. dros. hell. hydr. lact. puls. stann. stram. thea. thu.
— — left side only: phos.
— **pursued**, when walking, as if: *anac.*
— **restless**, driving one about: acon. alum. ambr. anac. arg. ARS. asaf. *aur.* bell. bov. bry. calc. calc-p. camph. caps. carb-an. carb-v. caus. *cham.* chin. chin-s. cimic. coff. croc. crotal. dros. graph. hell. hepar. lact. lam. men. merc. nat-c. nat-m. nit-ac. nx-v. op. phos. ph-ac. plat. puls. rhus ruta. sabad. sep. sil. spig. spong. staph. *tabac.* valer. verat. znc.
— — driving out of bed: *ars.* bry. carb-v. caus. *cham.* chin. chin-s. *graph.* *hepar.* hyos. nat m. nit ac.
— — compelling rapid walking: *arg-n.* ars. lil-t. sul-ac.
— **salvation**, about: graph. hura. lyc. puls. sulph. verat.
— **trifles**, over: anac. *ars.* bar-ac. calc. cham. chin. con. *ferr.*
— — morning: cocc.
— — evening, in bed: laur.
— — before menses: con.
— **undefinable**: all-c. merc.

**Conditions and Concomitants of Anxiety.**

— **morning**: ailan. alum. am-c. anac. ARS. canth. carb-an. carb-v. caus. chin. cocc. con. *graph.* ign. ipec. led. lyc. mag-c. mag-m. mezer. nat-m. nit-ac. *nx-v.* PHOS. plat. puls. rhus. sep. *sulph.* sul ac. verat. znc.
— — on waking: alum. anac. carb-an. carb-v. *caus.* chel. chin. graph. ign. ipec. *lyc.* mag-s. nat-m. nit-ac. *nx-v.* *phos.* plat. puls. rhus. sep. squil.
— — — at 3 A. M.: ARS.
— — after rising: arg-n. carb-an. mag-c. rhus.
— — — ameliorated on rising: nx-v.
— **forenoon**: acon. am-c. lyc. nat-m. pæon. plat ran-b. sars.
— **afternoon**: æth. am-c. bell. bov. calc. carb-an. gamb. mag-c. nat-c. ph-ac. puls. rhus. ruta. stront. tabac.
— — till evening: mag-m.
— **evening**: agar. alum. ambr. anac. ant-t. *ars.* bar. bell. borax. bov. bry. cact. calad. *calc.* carb-an. *carb-v.* caus. chel. chin. cina. cocc. dig. dros. fluor-ac. graph. *hepar.* hipp. k-ca. k-iod. lact. *laur.* *lyc.* mag-c. mag m. merc. mezer. mur-ac. nat-c. nitr. nit-ac. nx-m. *nx-v.* pæon. petr. *phos.* puls. ran-b. *rhus.* ruta. sabin. *sep.* sil. spig. *stann.* stront. SULPH. tabac. verat.
— — — aggravated in: *borax.* carb-an. dig. dros.
— — ameliorated in: am-c. chel. verb.
— — **in bed**: *ambr.* am-c. anac. ant-cr. ars. *bar.* *bry.* calad. *calc.* carb-an. *carb v.* caus. cham. *cocc.* *graph.* hepar. laur. *lyc.* *mag-c.* mag-m. nat-c. nit-ac. nitr. nx-v. phos. puls. sabin. sep. sil. stront. sulph. verat.
— — — uneasiness and anxiety, must uncover: mag-c.
— — in the **twilight**: ambr. ars. *calc.* carb-v. dig. laur. nx-v. *phos.* *rhus.* sep.
— — 4 to 6 P. M.: carb-v.

— **night**: *acon.* agar. *alum* ambr. am-c. am-m. ant-cr. arg. arn. *ars.* bar. *bell.* borax. bov. bry. cact. *calc.* camph. cann s. canth. *carb an.* *carb-v.* *caus.* *cham.* *chin.* chin-s. cina. clem. cocc. coff. con dig. dros. dulc. ferr. *graph.* *hæmat.* *hepar.* *hyos.* *ign.* jatr. k-ca. lact. lyc. mag-c. mag-m. mang. *merc.* merc-c. nat-c. nat m. *nit-ac.* nx-v. petr. *phos.* plat. plb. puls. ran-b. *rhus.* sabad. sep. sil. spong. squil. stront. sulph. *verat.* znc.
— — aggravated at: alum. carb-an. *rhus.*
— — on waking: alum. *ars.* carb-v. chel. con. graph. nat-m. nit-ac. *phos.* puls. SULPH.
— **midnight**, at, on awaking: ameliorated on rising: sil.
— — before: ambr. am-·. ars. bar. *bry.* *carb-v.* caus. *cocc.* *graph.* *hepar.* laur. *lyc.* *mag-c.* mag-m. nat-c. nx-v. phos. puls. sabin. sil. stront. *sulph.* verat.
— — after: alum. ant-cr. ARS. calc. chin. dulc. graph. lyc. mag-c. nx-v. *rhus.* squil.
— **abdomen**, during pain, in: æth. alum. am-m. ant-t. *ars.* *aur.* borax. bov. caps. cham. cupr. graph. k-clc. mezer. sep. *sulph.*
— — constriction in region of stomach: guai.
— — pressure in stomach: gent-l.
— — pressure at epigastrium: guai.
— — swelling of stomach: gins.
— — tension in, from: staph.
— **acids**, after: sulph.
— **air**, in open: acon. anac. arg. bell. cina. hepar. ign. lach. plat.
— — — — relieved: arund. *bry.* grat. laur. mag-m. rhus.
— **anger**, during: sep.
— **apparition**, from horrible, while awake: znc.
— **approach** of others, from: ambr. *arn.* iod. lyc.
— **ascending** steps, on: *nit-ac.*
— **back**, from pain in: junc.
— **breathing**, deeply, on: acon. *spig.*
— — — amel. agar. rhus.
— — with difficult, dyspnœa, etc.: acon. ambr. am-c. anac. ars. borax. calc. carb-v. cocc. creos. hepar. hydr-ac. iod. lact. lyc. nit-ac. nx-v plat. rhus. seneg. sil. spig. tabac. verat.
— **bed**, in: cham. mag-c. mag-m.
— — see under Morning or Evening.
— **beer**, after: ferr.
— **bronchi**, from mucus in: arund.
— **chagrin**, after: lyc.
— **chest**, during congestion of: sep.
— — constriction of: gins.
— — heat in, rising to mouth: nx-v.
— — — burning in: gels.
— — — warm orgasm of: nx-v.
— — pain in, with: rhus.
— — pressure on: sulph.
— — tension of: gins.
— **children**, in: borax. calc. k-ca.
— — when lifted from the cradle: calc-p.
— **chill**. See under Fever.
— **coffee**, after: bart. cham. ign. nx-v.
— — ameliorates: morph.
— **coition**, after: sep.
— — after pollutions: carb-an. petr.
— **cold** drinks, amel.: acon. *agar-em.* sulph.
— — with coldness: cup-ac. nit-ac.
— **company**, when in: *acon.* bell. lyc. petr. plat.
— — when alone: *dros.* mezer. *phos.* tabac. znc.
— — — — aggra. alcoh.
— **cough**, with: *acon.* cina. *coff.* dros. eup-per. *hepar.* iod. rhus. samb. spong. stram.
— — before attack of whooping-cough: cupr.
— — from coughing: merc-c. nit-ac. stram.
— **dancing**, when: *borax.*
— **diarrhœa**, during: crotal.
— — see also under Stool.
— **digestion**, during: ferr.
— **dinner**, during: mag-m.
— — after: ambr. canth. gins. hyos. nat-m. *phos.* sil. verat.
— — see also Eating.
— **dreams**, on waking from frightful: ars. chin.
— — see also Dreams, Anxious.
— **eating** before: mezer. ran-b.
— — when: carb-v. mag-c. mezer. sabad. *sep.*
— — after: ambr. asaf. canth. carb-an. carb-v. caus. chin. cocc-c. con. ferr-m. hyos. k-ca. lach. mag-m. merc. nat-c. nat-m. nit ac. nx-v. phel. phos. ph-ac. sep. sil. thu. verat. viol-tr.
— — supper or warm food, when: mag-c.
— — ameliorates: aur. mezer. sulph.
— — — after breakfast: k-ca.

— **ears,** with noise in: puls.
— — with sensitiveness to noise: caps.
— **eructations,** amel.: mag-m.
— **excitement,** from: asaf.
— **exercise,** amel.: tarent.
— **eyes,** from exerting the: sep.
— — with obscurations of: arg-n. staph.
— — with dilated pupils: nx-v.
— **face,** with hot: *acon.* arg-n. bell. carb-v. graph. merc.
— — — pale: puls.
— — — red: acon. sep.
— — — sweat on: ars. cic. mur-ac. nat-c.
— **fainting,** with: ars. cic. ign. mag-m. nit-ac. op. ran-b.
— **feet,** with cold: cupr. graph. sulph. thu.
— — after bathing the: nat-c.
— — with heaviness of: ign. nit-ac.
— **fever,** during prodrome: ars. chin.
— — during chill: acon. arn. ars. camph. caps. carb-v. chin. ign. nat-m. nx-v. phos. plat. puls. verat.
— — after chill: k-ca.
— — during heat or fever: ACON. *alum.* ambr. arn. ARS. asaf. bar. bell. berb. bov. *bry. calc.* canth. carb-an. casc. cham. chin. coff. crotal. cyc. dros. ferr. fluor-ac. graph. grat. hepar. hyper. ign. ipec. lach. *mag-c.* mag-m. merc. nat-c. nat-m. nicc. nx-v. op. phos. ph-ac. plan. plat. plb. *puls.* rheum. ruta. SEP. *spong.* stann. stram. sulph. verat.
— — during perspiration: ambr. am-c. ant-cr. arn. ars. bar. bell. benz-ac. berb. bry. calc. cann-s. caus. cham. cic. cocc. ferr. graph. mag-c. mang. *merc.* mezer. mur-ac. *nat-c.* nat-m. nit-ac. nitr. nx-v. phos. plb. puls. rhus. samb. *sep.* spong. stann. staph. stram. sulph. thu.
— — — sweat amel. anxiety: agar.
— — during apyrexia: acon. camph.
— **fingers,** starting in: puls.
— **fits,** with: alum. bell. caus. cupr. cocc. ferr. hyos. ign.
— **flatus,** from: coff. *nx-v.*
— — emission of, amel.: calc-ac.
— **fright,** after: sil.
— **gastric** complaints, with: alum. am-m. ars. bar-m. calc. cupr. nit-ac. nx-v. phos. puls.
— **hands**, cold, with: graph. puls.
— — with hot: *carb-v.*
— — with sweat on: cham. merc.
— — with trembling of: am-c. cic. plat. puls.
— **headache,** with: acon. alum. bell. bov. calc. carb-v. caus. graph. laur. mag-c. phos. puls. ruta. sulph.
— **head,** with congestion to: *acon.* carb-v. laur. *mag-c. phos. puls.* sulph.
— — with dullness of: alum. bov.
— — with heat of: carb-v. laur. *mag-c. phos.* sulph.
— — with pressure from forehead to vertex: glon.
— — with stupefaction of: alum. bov. caus. sil.
— — with sweat on: ars. carb-v. nx-v. phos. sep.
— **heart,** palpitation of, causes: graph. sep. sulph. trill. *verat.*
— — with pain at: bar. carb-v. cham. nit-ac. nx-v. rhus. spong.
— — with palpitation of: *acon.* alum. am-c. ant-t. aspar. ars. aur. borax. calc. cann-s. carb-v. caus. cham. *dig.* ferr. ign. k-ca. lach. lyc. mosch. nat-c. nat-m. nit-ac. nx-v. olnd. phos. plat. plb. *puls.* ruta. sars. sep. sil. *spig.* verat. viol-od. viol-tr.
— — with pressure at, tearing at loins and restlessness: rhus.
— — during pain at region of: hæmat.
— — during throbbing in cardiac region: graph.
— **heat.** See Fever.
— **hypochondriacal**: am-c. arn. ars. asaf. calad. canth. cham. dros. k-clc. mosch. nat-m. nit-ac. phos. ph-ac. valer.
— **hysterical**: asaf. con.
— **house** or room, in: aster. bry. chel. k-ca. mag-m. *rhus.* tilia. valer.
— — on entering: rhod.
— — ameliorated in: ign.
— **hungry,** when: k-ca.
— **lameness,** with: aloe.
— **legs,** with pains in: borax. rhus. sars.
— — starting in: hepar.
— **light,** in candle: bov.
— — ameliorates: nat-m.
— **limbs,** with heaviness in: mag-c.
— — with pain in: ars. bell.
— — with trembling in: ars. aur. calc. carb-v. caus. *cham.* coff. croc. cupr. graph. *lach.* mag-c. mezer. mosch. *nat-c.* phos. puls. rhus. sars. sep.
— **loins,** from aching in, after supper: nx-v.

— **lying** down, when : carb-v. sil. stann.
— — has to lie down, with anguish: mezer. phel.
— — evening. after: hepar. nx-v.
— — — See Evening in Bed.
— — on side: bar. k-ca. phos. puls.
— — on left side: bar. phos.
— — on right side: k-ca.
— **meal**. See Eating.
— **menses**, before: acon. am-c. carb-v. *cocc.* con. k-bi. mang. merc. *nat-m.* *nit-ac.* *nx-v.* stann. sulph. znc.
— — **during**: acon. *bell.* calc. canth. cimic. cina. coff. con. ign. k-iod. merc. *nat-m.* nit-ac. nx-v. phos. plat. *sil.* stann. sulph. znc.
— — amel.: stann. znc.
— — **after**: *agar.* phos. secale.
— — which prevents sleep: agar.
— **mental** labor, from: nat-c. plan.
— **meditation**, from: acon. ars. calc. camph. cham. nx-v. puls. rhus. secale. verat.
— **micturition**, during: cham.
— **moaning**, with: acon. alum. ant-t. ars. cham. par. phos. rheum.
— **motion** from: acon. berb. borax. mag-c. nicc. rheum.
— — aggra.: nat-c.
— — ameliorates: sil.
— **nausea**, with: alum. am-m. bar-m. *calc.* graph. nit-ac. nx-v. puls. tabac.
— **noise**, from: agar. aur. caus. chel. *sil.*
— **pains**, from the: *acon.* *ars.* caus. nat c.
— **periodical** attacks of: arn. ars. *cham.* cocc. nat-c. nat-m. phos. plat. sep. spong. *sulph.*
— **reading**, while: mag-m.
— **rest**, when at: acon. seneg.
— — in bed aggr.: alcoh.
— **retching**, with: bar-m.
— **riding**, when: borax. lach. psor.
— — down hill: *borax.*
— **rising**, after: chel. mag-c.
— — see also under Morning.
— — from a seat, on: verat.
— — — — — amel.: mill.
— **screaming**, with: calc. chin-s. cocc. hyos. lyc. ran-sc.
— **sedentary** employment, from: ars. graph.
— **shaving**, when: calad.
— **shuddering**, with: bell. calc. carb-v. nat-c. plat. puls. tabac. verat.
— **sitting**, when: caus. *graph.* nit-ac. phos. tarax.
— — aggravates: sil.
— — cannot sit on account of anguish: graph. sil.
— — when at work, while: graph.
— — bent over, anxiety with fear of death: rhus.
— — — — with pain in rectum, has to walk: calc.
— — still, all anxiety disappears: iod.
— **sleep**, before: alum.
— — in evening until he goes to sleep: berb.
— — on going to: calc. lyc. *puls.*
— — during: cocc. graph. lyc. nat-m. nx-v. phos. spong. verat.
— —on starting from: clem.
— — during partial slumbering in morning: junc.
— **sleepiness**, with: ars. borax. led. nx-v. rhus.
— **sleeplessness**, with: acon. agar. arn. *ars.* bar. *bell.* bry. carb-an. carb-v. caus. *cham.* chin. chin-s. cocc. *coff.* con. cupr. ferr. *graph.* *hepar.* *hyos.* k-ca. laur. mag-c. *merc.* nat-c. nat-m. nit-ac. ran-b. ran-sc. sabin. sep. sil. sulph. thu. verat.
— **soup**, after: mag-c. ol-an.
— **speaking**, when: alum. ambr. plat.
— — aggravates: nat-c.
— — on attemping to talk in company: plat.
— **speechlessness**, with: ign.
— **standing**, when: sil. *verat.*
— — amel., when: calc. phos. tarax.
— **start**, from a: *sil.*
— **stool**, before: ars. cadm. cham. croton. merc.
— — during; cham. merc. sulph. verat.
— — after: acon. *caus.* carb-v. k-ca. *nit-ac.*
— **stooping**, when: rheum.
— — great anxiety, amel. by stooping: bar-m.
— **storm**, during a: gels. *nat-c.* nat-m. *nit ac.* phos.
— **sudden**: *cocc.* plat. thu.
— **supper**, after: mag-c. nx-v.
— **sweat**. See under Fever.
— **taste**, with bitter: am m. bell.
— **thinking** about it, excited by: alum. ambr. bry. *calc.* caus. con. *nit-ac.* staph. tabac.
— **thirst**, with: cup ac.
— **throat**, with dryness of: rhus.

— **tobacco**, from smoking: petr. sep.
— **tremor**, with: ant-t. ars. cham. euph. mag-c. plat. *puls*. sars.
— uneasiness and anxiety at night, must uncover: mag-c.
— **vertigo**, with: bar m. bell. caps. caus. coff. ign. merc. nx-m. nx-v. op. rhod. rhus. seneg. sep. sil. verat.
— — after: aloe. gamb.
— **vexation**, after: lyc. sep. verat.
— **voice**, on raising the: cann-s.
— **vomiting**, before: arn. sang.
— — ameliorates: hell. tabac.
— — during: ars. ars-h. cup-ac. gran. nit-ac. seneg. tabac. tong.
— — after: k-ca.
— **waking**, on: acon. am-c. cact. calc. carb-v. caus. chin con. dros. graph. ign. ipec. lyc. nit-ac. nx-v. phos. plat. puls. rhus. samb. sep. sil. sol-t-æ. *sulph*.
— — from a dream: graph. sil.
— — see also under Morning and Night.
— **walking**, on: anac. arg. arg-n. bell. cina. clem. hepar. ign. nx-v. plat. staph. tabac.
— — rapidly, when: nit-ac. staph.
— — amel., in open air: rhus.
— — aggra.: plat.
— — in open air: anac. arg-n. bell. cina. hepar. ign. nx-v. plat.
— — in cool air: nx m.
— **warmth**, afternoon, during: gamb.
— **weakness**, with: acon. agn. alum. *am-c*. ang. ars. *aur*. borax. calc. carb-an. carb-v. caus. cic. ign. mag-c. nitr. phos. rhus. sil. verat.
— **weeping**, followed by: acon. am-m. carb-v.
— — amel.: dig. graph. *tabac*.
— **work**, during: anac. graph. iod.
— — had to stop, for anxiety: aloe.
— **yawning**, with: plb.

**Anxiousness**. See Anxiety.
**Apathy**. See Indifference.
**Apprehensions**. See Fear, Anxiety, etc.
**Arrogance**: alum. arn. chin. cic. cupr. ferr. hyos. ipec. *lach*. lyc. par. PLAT. *stram verat*.
**Asking** and then refusing. See Refuses.
**Asks** for nothing: bry. puls. rheum.
**Attention**, active: coff. oxal-ac.
— difficult to fix (distraction, etc.): acon. æsc-h. *agn*. ailan. all-c. alum. am-c. anac. ang apis. arn. ars. bar. bell. bov. camph. cann-i. cann-s. canth. carb an. caus. cham. chel. chin-s. *cimic*. cinnb. cocc. coff. colch. coloc. corn. croc. cup-ac. elaps. ferr. fluor-ac. *gels*. glon. graph. ham. hell. hura. hydr. hydr-ac. hyos. ign. iod. iris. k-bro. k-ca. kalm. lach. lam. laur. lil-t. mag-c. mang. merc. merc-c. mezer. mosch. nat-c. nat-m. nx-j. nx-m. nx-v. olnd. ol-an. op. phos. ph-ac. phys. plat. poth. puls. ran-sc. rhod. rhus. rhus-r. sars. senec. sep. *sil*. spig. stann. staph. stram. sulph. sul ac. tabac. *tereb*. thu. verb. viol-od. znc.
— **want**, of (inattention): alum. am-c. ang. bar. bell. bov. caus. cham. cimic. k-ca. merc. nat-c. nat-m. nx-v. olnd. ph-ac. plat. puls. rhod. *sep*. sulph. thu.-tilia.
— — when learning, reading, etc.: alum. asar. *bar*. bell. caus. cham. coff. k ca. nat-c. spig. sulph.
— — in children: *bar*.

**Audacity**: op.
— compare with Boldness, etc.
**Avarice** (covetousness, etc.): ars. bry. calc. cina. coloc. nat-c. *puls*. rheum. *sep*.
**Aversion**, takes involuntary, to certain persons: am-m.
— in general. See name of thing disliked.
**Awkward**: æth. agar. ambr. anac. *apis*. asaf. asar. bov. camph. *caps*. cocc. ign. ipec. lach. *nat-c*. *nat-m*. *nx-v*. plb. *puls*. sabin. sars. sil. spong. stann. stram. sulph. thu.
— afternoon: anac.
— evening: agar.
— breaks things: apis.
— in children during headache: caps.
— things fall from one's hand: *apis*. bov. bry. hell. hyos. nx-v.
— in dressing, dresses unbecomingly: hyos. stram.
— knocks against things: caps. ipec. nat-m. nx v. op.

**Barking**: bell. canth. stram.
**Bashful** (diffident, timid): aloe. aur. bar-ac. bell. carb-an. *chin*. con. coff. *ign*. iod. k-bi. *nat-c*. nit-ac. nx-v. phos. stram. sulph. tabac.
— after a fright: *acon*.
**Bed**, aversion to (shuns): cann-s. caus lyc. nat-c.
— — great dread of going to bed: cann-s.
— desires to remain in: merc. psor.

— goes from one bed to another, etc.: ars. hyos.
— see also under Delirium.
**Begging**, entreating: ars. stram.
— in sleep: stann.
**Bellowing**: bell. *canth.*
**Bemoaning**. See Lamenting.
**Bewildered**: acon. *coff.* colch. nx-v. puls. stram. valer. verat.
— during paroxysms of pain: acon. cham. *coff.* verat.
— loses way in well-known streets: glon. nx-m. petr
— on waking does not know where she is: æsc-h.
— — — knows no one; is terrified: stram.
**Biting**: *bell.* bufo. canth. cic. croc. cupr. hydrph. hyos. hura. secale. ***stram.*** verat.
— fingers: arum-t. plb. op.
— hands: hura.
— pillow: phos.
— himself: tarent.
— tumbler: ars.
— spoons, etc.: bell.
**Blindness**, pretended: verat.
**Bliss**, feeling of: op.
— see Happy, etc.
**Blood**, cannot look at, on a knife: alum.
**Boldness**, courage: acon. agar. alum. ant-t. berb. bov. calad. dros. gins. *ign.* merc. mezer. nat-c. *op.* phos. puls. squil. sulph. tarax. valer. verat.
**Break**, desires to, everything: hura.
**Brooding** mood: alum. arn. aur. canth. caps. caus. cham. cyc. euphr. hell. ipec. mezer. mur-ac. nx-v. olnd. op. sulph.
**Business**, averse to: brom. fluor-ac. k-ca. puls. sep.
**Calmness**: op. verat.
**Calumniate**, desire to: ipec.
**Capriciousness**: acon. alum. am-c. arn. *ars. bell.* bry. calc. caps. carb-an. cham. chin. cina. creos. croc. dig. hepar. ign. k-ca. lyc. merc. nit ac. nx-v. phos. puls. secale. sil. spong. stram. sulph. viol-tr.
**Carefulness**: iod. nx-v. puls.
— See also Scrupulous.
**Carelessness**: aur-m. gels. op. verat.
**Cares**, full of: caus. *chin.* coff.
— about domestic affairs: bar. puls. sep.
— about trifles: *ars.*
— indifferent to: nit-ac.
**Carried**, desires to be: acon. ant-t. ars. brom. *cham.* cina. ign. lyc. k-ca. puls. verat.
— — — in croup: brom.
— — — fast: ars.
— — — slowly: puls.
**Cautious**: graph.
**Censorious**, critical: acon. alum. arn. *ars.* aur. bell. borax. calc-p. caps. caus. cham. chin. cic. gran. guai. hyos. ign. ipec. *lach.* lyc. merc. mezer. mosch. nat-m. nx-v. par. petr. plat. rhus. *sep.* staph. sulph. tilia. verat.
— disposed to fault finding or is silent: *verat.*
**Changeable** humor. See Humor.
— everything seems changed, after short absence: plat.
**Chases** imaginary objects: stram.
**Cheerful** (gay, happy, jolly, etc.): acon. æsc-h. æth. agar. anac. ang. apoc. *arg.* ars. asaf. bell. borax. bov. brom. bry. cann-i. cann-s. canth. caps. carb-v. caus. chin. cic. coca. cocc. coff. colch. coloc. *croc.* cupr. cyc. dros. eucal. ferr. fluor-ac. gamb. gels. hydr. hyos. *ign.* k-bi. k-bro. lach. laur. lyc. manc. marum. merc. nat-m. nat-s. nit ac. ol-an. op. oxal-ac. petr. phos. ph-ac. phys. plat. plb. psor. rhod. ruta. sabad. sars. secale. seneg. sep. spig. spong. squil. stann. stram. tarax. tarent. therid. thu. valer. verat. verb. viol-od. znc. zing.
— **alternating**, with aversion to work: spong.
— — with ill humor: ant-t. aur. chin. cyc. lyc. merc. nat-c. nat m. plat. spig.
— — with indifference: tarent.
— — with mania: bell. cann-s. croc.
— — with melancholy: asar. chin. ferr. znc.
— — with pain: plat.
— — with passion, burst of: aur. caps. croc. ign. stram.
— — with sadness: acon. asar. canth. carb-an. caus chin. clem. croc. ferr. fluor-ac. gels. ign. iod. lyc. nat-c. nit-ac. nx m. plat. psor. senec. sep. spig. tarent. znc. ziz.
— — with vexation: ant-t. borax. caus. cocc. croc. spong. znc.
— — with violence: aur. croc. stram.
— — with want of sympathy: merc.
— — with weeping: acon. arg. bell. borax. cann-s. carb-an. ign. iod. plat. spong.

— **followed** by crossness, etc.: clem. hyos. nat-s. ol an. op. seneg. tarax.
— — — melancholy: gels. graph. petr. plat. ziz.
— — — prostration, etc.: clem. spong.
— — — sleepiness: bell. calc.
— **conditions** of cheerfulness.
— **morning**: borax. caus. fluor-ac. hura. lach. nat-s. plat. sulph.
— — on waking: aloe. clem. hydr. tarent.
— **evening**: agar. aloe. bell. bism. chin s. cupr. cyc. ferr. hydrph. lach. marum. merc i-fl. merc-i-r. nx-m. ol-an. valer. viol-tr. znc.
— **night**: op. verat.
— **air**, in open: merc-i-fl. tarent.
— — — walking: alum. ang. cinnb. plb. tarent.
— **eating**: bell. cist.
— **menses**, before: acon. fluor-ac. hyos.
— — during: *fluor-ac.* stram.
— **room**, in the: amel. tarent.
— **stools**, after: borax. nat-s. oxal-ac.
— **supper**, after: cist.
**Childish** behavior: acon. anac. bar. carb-an. carb-v. cic. *croc. ign.* nx-m. par. seneg.
— see also Foolish.
**Children**, aversion for: raph.
— desires to beat: chel.
— — to have, to beget: oxal-ac.
**Clairvoyance**: acon. calc. cann-i. hyos. phos. sil. stann.
**Clinging** to persons or furniture: coff.
— child awakens terrified, knows no one, screams, clings to those near: stram.
**Clothed** improperly: hell. hyos. stram.
**Cloudiness**, confusion. See Stupefaction.
**Coldness**, of disposition: plat. sabad. squil.
**Color**, aversion for blue: tarent.
**Company**, averse to (desires solitude): acon. *ambr.* alum. anac. ant-t. atrop. *aur. bar.* bell. bufo. *cact.* calc. calc-p. camph. cann-i. *carb-an.* carb-v. chin. cimic. cinnb. cic. clem. coloc. con. cupr. cyc. *dig.* dios. elaps. eugen. ferr. fluor-ac. gels. graph. grat. hell. hepar. hydr. hyos. ign. k-bi. k-ca. lach. led. lil-t. lyc. mag-m. mang. men. *nat c.* nat-m. nicc. nx-v. petr. phos. pic-ac. *plat.* puls. rhus. secale. sep. stann. sulph. sul-ac. thu.
— — amel. when alone: bar. lyc. plb. *sep.*
— — averse to having any one near: sulph.
— — — to meeting friends, whom he imagines he has offended: ars.
— — — to presence of strangers: *ambr.* bar. lyc. petr. *sep. stram.*
— — avoids the sight of people: cupr.
— — during hot stage of fever: con. hyos. *puls.*
— — during sweat: ars. *bell.* lach. lyc. puls. sep.
— — during menses: plat. sap.
— — — — desires to be let alone: cic. nx-v.
— — when pregnant: lach.
— **desire** for (aversion to solitude): ant-t. *ars.* bism. bov. calc-p. dros. elaps. hyos. ign. k-ca. lac-can. lil-t. lyc. *mezer. phos.* ran-b. sep. *stram.* strych. verat. verb. znc.
— — aggr. when alone: brom. elaps. phos. stram.
— — of a friend, evening: plb.
— — during menses: stram.
**Complaining**: acon. ars. *bism.* bry. chin. *cina.* cocc. dig. hell. ign. k-iod. lyc. *nx-v.* op. petr. puls. *sulph.* tarent.
— alternating with delirium: bell.
— in sleep: bell. ign.
— on waking: cina.
— of supposed injury: hyos.
— of his illness: ph-ac.
**Comprehension**. See Memory.
— difficult. See Mind, weakness of, etc.
**Conceals** himself: *ars.* bell. cupr. *hell. puls.* stram.
**Concern**, little as to his health: cocc.
— see Fear, Anxiety, etc.
**Condescension**: ars. croc. ign. lyc. mosch. *puls.* sil.
**Confidence**, want of self: anac. ang. aur. bar. bell. calc cic. canth. chlor. dros. hyos. ign. lach. lyc. merc. nat-c. olnd. op. puls. rhus. ruta. stram. sul-ac. therid.
— — — and thinks others have none, which makes her unhappy: aur.
— — — in his own strength: agn. ang. canth. nx-v. olnd. viol-tr.
**Confounding** objects and ideas: calc. cann-s. hyos. nx-v. plat. *sulph.*
**Confusion**. See under Head and compare Bewildered, etc.
— of ideas. See under Thoughts.
**Conscience**, remorse of: alum. am-c. *ars. aur. bell.* carb-v. caus. cham. cina. *cocc.* coff. con. *cupr.* cyc. dig. ferr. graph.

hyos. *ign.* lach. merc. nat-m. nit-ac. nx v. puls. ruta. sabad. selen. *sil.* stram. stront. sulph. *verat. znc.*
— — about trifles : *sil.*
— — **afternoon** : am-c. carb-v.
— — **night** : puls.
— — **waking**, on : puls.
— — **debility**, with : am-c.
— — trembling, with : carb-v.
— — — see also under Delusions and Anxiety.
**Conscientious** : *ars.* cham. cyc. hyos. *ign.* iod. *lyc.* nx-v. puls. sil. stram. *sulph.* thu. verat.
**Contemptuous** humor : alum. *ars. chin.* cic. guai. ign. ipec. lach. *lyc.* nat-m. *nx-v.* par. *plat.* puls. spong.
— — worse in open air or when sun shines into room : plat.
— — for everything : chin. cina. ipec.
— — for self : agn. cop.
— — in paroxysms, against her will : plat.
**Contented** : aloe. aur. caps. cic. coca. cocc. fluor-ac. gins. mag-s. men. mezer. nat-c. op. spig. tarax. znc.
— see also Cheerful.
— after stool : *borax.*
**Contradict**, disposition to : anac. arn. aur. camph. canth. *caus.* ferr. grat. hyos. ign. lach. lyc. merc. nat-c. nicc. nx-v. *olnd.* poth. ruta.
— is intolerant of : am-c. *aur.* cann-i. ferr. helon. ign. lyc. nicc. *nx-v.* olnd.
— — — has to restrain himself to keep from violence : sil.
**Contrary** humor, everything is disagreeable : acon. alum. ambr. ant-c. ars. aur. bell. calc. caps. caus. con. croc. hepar. ign. ipec. lact. led. mag-c. mag-m. *merc.* nit ac. nx-v. petr. phos. plb. *puls.* samb. sars. sil. spong. sulph. trom.
— compare Ill-Humor, etc.
**Conversation**. See Talking.
**Counting**, continually : phys.
**Courage**. See Boldness.
**Covetous**. See Avarice.
**Cowardice** : *acon.* agar. agn. alum. anac. ang. aur *bar-ac.* bell. *bry.* calc. camph. canth. carb-v. caus. *chin.* cocc. coloc. con. cupr. dig. dros. graph. ign. iod. ipec. k-ca. laur. led. lyc. merc. mur-ac. nat-m. nit-ac. nitr. op. phos. ph-ac. plb. *puls.* ran-b. ruta. sabin. secale. sep. sil. spig. stann. sulph. sul-ac. tabac. thu. verat. verb. viol-tr.
**Crazy**. See Insanity, and under Delusions, etc.
**Criminal**, thinks he is a : cob. cyc. dig. hyos.
— — others know it : cob.
— see Delusions.
**Critical** mood. See Censorious.
**Croaking** : cina. cupr. cup-ac.
— in sleep : bell.
**Cross**. See Fretful, Ill-Humor.
**Cruelty** : anac. croc. op.
— desires to be cruel : abrot.
— compare Unfeeling.
**Crying** out : alum. ant-c. arn. ars. bell. *borax.* calc. caus. *cham.* chin. cina. ipec. samb. sep. sil. verat.
— — during dreams : fluor-ac.
— — incoherent and rapid exclamations : stram.
— — suddenly : *apis.* ars. glon. hell. *hyos. lyc.* rhus. *stram.*
— — feels as though she must scream : anac. calc. elaps. lil-t. nx-v. sep. sil.
— compare with Screaming, Weeping.
**Cursing** : *anac* ars. bell. borax. bov. cann-i. coral. lil-t. lyc. nit-ac. nx-v. op. plb. puls. *verat.*
— desire to : lil-t.
— during convulsions : ars.
— irresistible desire to curse and swear : ANAC.
— all night and complains of stupid feeling : verat.
**Cut** others, desires to : hydrph.
**Dancing** : acon. agar. apis. *bell.* cann-i. cic. con. croc. grat. hyos. nat-m. ph-ac. plat. *stram.* tabac. tarent.
— alternating with sighing : bell.
— desires to : croton. nat-m.
— grotesque : *cic.*
— unconscious : ph-ac.
— wild : tarent.
**Dark**, mentally worse in the : calc. carb-an. caus. plat. rhus. *stram.* valer.
**Deafness**, pretended : verat.
**Death**, fear of : *acon.* agn. alum. am-c. anac. *apis. ars.* bar. *bell.* bry. calc. caps. caus. cocc. cupr. dig. graph. hepar. ipec. k-ca. *lach.* lyc. *mosch.* nitr. nit ac. nx-v. op. *plat.* puls. rhus. secale. squil. stram. verat. znc.
— — — during hot stage : calc. cocc. ipec. mosch. *nit-ac.* ruta.
— — — during sweat : nitr.
— — — during menses : acon. plat. verat.
— — — of sudden death : ars.

— **desires**: agn. apis. *aur.* carb-v. chel. clem. creos. der. gad. hura. lil-t. *merc.* mezer. nat-s. nit-ac. op. phyt. psor. *sil.* verat-v. vip.
— — during convalescence: absin. aur. lac-can. sep.
— — evening: aur.
— **presentment** of: *acon.* agn. alum. ars. bapt. *bell.* calc. canth. cimic cupr. k-ca. *lach.* lyc. merc. mosch. nat-m. nitr. nit-ac. *nx v.* phos. *plat.* sep. staph. stram. verat. znc.
— — — predicts the time: *acon.*
— — — thinks of death calmly: znc.
— — — believes that she will die soon and that she cannot be helped: *agn.*
— **sensation** of: æsc h. *agn.* camph. cann-i. cic. *graph.* k-bi. nitr. nx-v. op. *plat.* sil. verat.
— — — during chill: cann-i.
— — — during spasm: nx-v.
— **thoughts** of: agn. stram. verat.
**Deceitful**: coca. dros.
**Deeds**, feels as if could do great: hell.
**Defiant**: acon. anac. *arn.* canth. caus. guai. *lyc.* nx-v. spong.
**Dejection**. See Sadness.
**Delirium**: absin. acet-ac. *acon.* æsc-h. æth. agar. alcoh. am-c. anac. ant c. ant-t. arn. *ars.* aur. bapt. *bell.* *bry.* calad. cact. calc. camph. cann-i. cann-s. canth. carb-v. *cham.* chin. chin-s. *cic.* cimic. *cina.* coff. colch. coloc. con. crotal. croc. *cup-ac.* cupr. dig. dulc. ether. glon. hell. hipp *hyos.* ign. iod. jatr. k-bro. k-ca. lach. lachn. lact. lil-t. lyc. *meli.* men. merc. *merc-c.* merl. mezer. mur ac. nitr. nit ac. nx-m. nx-v. *op.* o[illegible]-ac. petr. *phos.* ph-ac. plat. *plb.* podo. psor. puls. ran-b. ran-sc. rheum. rhod. *rhus.* sabad. sabin. sal ac. samb. *secale.* *stram.* *sulph.* sul-ac. tarax. taxus. thea. valer. *verat.* *verat-v.* vesp vip. znc.
— Compare with Speech.
— **abandons** her relatives: secale.
— **absurd** things, does: secale.
— **alternating** with colic: *plb.*
— — with coma, also with somnolency: plb. stram.
— — with sopor: acet-ac. cocc. plb. vip.
— — — tetanic convulsions, lies on his back, knees and thighs flexed, hands joined: *stram.*
— **antics**, plays: lact.
— **anxious**: acon. anac. bell. camph. hepar. hyos. nx-v. op. phos. sil. stram. verat.
— **apathetic**: verat.
— **arms**, throws about: bell.
— **attacks** people with knife: hyos.
— **barking**: bell.
— **bed**, attempts to leave. See Escape.
— — creeps about in: stram.
— **biting**: bell. bufo. hyos. hydr-ac. secale. stram.
— **blames** himself for his folly: op.
— **blessing**, asks a: bell.
— **busy**: bell. camph. hyos. stram.
— **business**, talks of: bry. op.
— **catches** at flocks in air: hyos. lyc. ph-ac. znc.
— — waves hands in air: op. stram.
— — reaches out after objects: psor. sulph.
— — plays with hands: hyos.
— see also Gestures.
— **cheerful**: acon. *bell.* cact. con. op. sulph. verat.
— — see also Laughing.
— **confused** talking: bell.
— **constant**: bapt.
— **crying**: bell.
— **disconnected** things, says: op.
— **dogs**, talks of: bell.
— **embraces** the stove: hyos.
— **erotic**: cann-i. phos. stram.
— **escape**, attempts to: alcoh. *bell.* cupr. dig. merc. phos. *stram.* sul-ac. verat.
— — — to leave bed: acon. alcoh. atrop. *ars.* *bell.* *bry.* chin. cic. gal-ac. *hyos.* merc. merc-c. op. phos. plb. sol-m. *stram.* sul-ac.
— — — continually leaving and returning to bed: bell.
— — — from her family, children: lyc.
— — — to run away: bell. bry. hyos. stram. op. stram.
— — — to change beds: ars. hyos.
— — — springs up suddenly from bed: bell. nx-v.
— — — is restrained with difficulty: znc.
— — — to escape from window: valer.
— — — wants to visit his daughter: ars.
— **faces**, sees strange faces, on closing eyes: ars. calc. carb-v. chin. samb.
— **fantastic**: *bell.* cham. graph. hyos. op. sep. sil. spong. *stram.* *sulph.*
— **feces**, swallows his own: verat.

— — licks up cow-dung, mud, saliva: merc.
— **fierce**; agar. bell.
— **fight**, wants to: bell. hyos.
— — tries to kill people: secale. stram.
— **foolish**, silly; acon. æth. bell. merc. stram.
— **foreign** countries, talks of: cann-i.
— — language, talks in a: *stram.*
— **frightful**: acon. anac. atrop. *bell.* calc. colch. coloc. hyos. nx-v. op. phos. puls. sil. *stram.* verat. znc.
— **fur**, wraps up in, during summer: hyos.
— **gather** objects off the wall, tries to: bell.
— **gay**: agar. *bell.* cann-s. con. hyos. lact. *stram.*
— — alternating, of laughing, singing, whistling, crying, etc.: stram.
— — — with melancholy: agar.
— **gestures**, making: ars. hyos. mosch. nx-v. puls. stram.
— **groping** as if in the dark: plb.
— **home**, prepares to go; bell.
— — talks of: bell. bry.
— — thinks is not at: bry. op. verat.
— **hysterical**, almost: bell.
— **incoherent**: bell. *lach. rhus.* stram.
— **intoxicated**, as if: carb-an. hyos. vip.
— **kisses** every one: *verat.*
— **knocking** against walls: apis. con.
— — face with fists: bell.
— **jumping**: acon. bell. lact. merc.
— **laughing**: acon. bell. lact. op. sep. stram. sulph. thea. verat.
— **loquacious**: agar. bapt. bell. cupr. lach. op. petr. plat. phos. plb. rhus. stram. verat.
— — indistinct: apis. hyos.
— **maniacal**: acon. æth. ailan. ant-cr. ars. bell. camph. cann-i. canth. chin-s. coff. colch. con. crotal. cupr. hell. hyos. indg. led. lobel. lyc. merc. merc-c. nx-m. œna. op. plb. rhod. secale. stram. tarent. tereb. verat.
— **merry**. See Gay.
— **mouth**, moves lips as if talking: bell.
— — puts stones in: merc.
— **murmuring**: arn. hyos. lyc. rhus. stram.
— — slowly: ph-ac.
— — to himself: hyos. tabac.
— **muttering**: ailan. bapt. bell. crotal. hyos. merc. nx-v. op. stram. tarax. verat.
— — to himself: bell. rhus.
— — in sleep: ars.
— **naked**, strips himself: bell. hyos. phos.
— **noisy**: bell. hyos. stram.
— **nonsense**, with eyes open: hyos.
— **paroxysmal**: bell. con.
— **periodic**: samb.
— **picking** at nose or lips, with: *arum-t.*
— **pulling** at bed-clothes: arn. ars. bell. colch. hyos. lyc. op. phos. psor. rhus. stram. znc.
— **quiet**: cup-ac. hyos. phos. plb. secale. tabac.
— **raging**, raving: acon. æth. agar. alcoh. ant-t. arg-n. ars. *bell.* bry. calc. camph. cann-i. *canth.* cic. chel. chin. cimic. cina. colch. coloc. cupr. dig. dulc. ether. glon. graph. hell. *hyos.* hyper. jatr. lobel. *lyc.* merc. mosch. nx-m. *œna. op.* par. phos. plb. puls. rheum. *secale.* sol-n. *stram.* sulph. sul-ac. tabac. tarent. verat. vip. znc.
— — alternating with consciousness: acon.
— — — with religious excitement: agar.
— — when aroused: phos.
— — in sleep: cup-ac. mur-ac.
— — during convulsions: ars.
— **rambling**: atrop. *bell.* plb.
— **recognizes** no one: merc. stram.
— — does not know his friends: bell. hyos. nx-v. op. stram. tabac. verat.
— **reproachful**: lyc.
— **religious**: alcoh. aur. *verat.*
— **restless**: acon. atrop. plb.
— **roaming** in fields, thinks is: rhus.
— **rocking** to and fro: bell. hyos.
— **rolls** on floor: op.
— **romping** with children: agar.
— **running** about: bell. con. hell. stram. sulph. verat.
— — away: bell. cupr. dig. nx-v. verat.
— — about and knocks head against wall: con.
— **same** subject all the time: petr.
— **scolding**: merc. stram.
— **screaming**: atrop. bell.
— **singing**: bell. cann-i. cocc. cupr. hyos. lact. stram. verat.
— — alternating with laughing, whistling, crying, etc.: stram.
— **silent**: agar. secale.

— **soliloquizes** much: rhus.
— **sorrowful**: acon. bell. dulc. lyc. puls.
— **spits** at those about him: canth. cupr. hyos.
— — and licks it up or rubs it over the floor: merc.
— **strikes**, attempts to: bell. canth. hyos. lyc. stram.
— **sudden**: stram.
— **talking** loud: bell. hyos. stram.
— — in rhyme: thea.
— — to herself: merc.
— — See Loquacious.
— **tremens**. See Mania-a-potu.
— **urinate** on the floor, tries to: plb.
— **visions**: bell. berb. bry. carb-v. dulc. hell. hepar. *hyos.* k-ca. mag-m. nat-c. nit-ac. nx-v. *op.* phos. *samb.* *stram.* sulph.
— — frightful: *bell.* bry. op. puls. stram.
— **violent**: atrop. *ars.* hyos. *op.* secale. *stram.*
— — displaying great strength: *agar.*
— — is restrained and calmed with great difficulty: znc.
— — at night: dig.
— — compare with Raving, etc.
— **vivid**: bell. *stram.*
— **vociferous**: stram.
— — see also Loquacious, Noisy.
— **wandering**. See Escape.
— **water**, jumping into: bell. secale.
— **wedding**, prepares for: *hyos.*
— **wild**: colch. hydr-ac. hyos. *stram.*
— — at night: gal-ac. plb.
— **wrongs**, of fancied: hyos.

**Conditions** of delirium.
— **evening**: *bell.* canth. croc. phos. plb.
— — during nap: nx-v.
— **night**: acon. ars. bell. *bry.* cact. cann-i. canth. codein. colch. crotal. dig. dulc. ether. graph. hepar. jabor. lyc. merc. merc-c. merc-sul. nx-v. op. plb. stram.
— — worse at night: ars. plb.
— **coldness**, with: verat.
— **convulsions**, during: ars. crotal.
— — after: absin. secale.
— **drunkards**, of. See Mania-a-potu.
— **eating**: amel. bell.
— **epilepsy**, during: op.
— — after: plb.
— **eyes**, on closing: bapt. calc. lach.
— — with open: ars. bapt. bell. cham. coff. coloc. *hyos.* op. stram. verat.
— **fatigue**, study, etc., from: lach.
— **fear** of men, with: plat.
— **feet**, with cold: znc.
— **fever**, during: æth. ailan. ars. bufo. chin. morph. sulph. sul-ac.
— — without fever: samb.
— **headache**, during: acon. agar. ars. glon. mag-c. mosch. secale. tarent. verat.
— **hemorrhage**, etc., after: china.
— **menses**, during: bell. hyos. lyc. nx-m. stram. verat.
— **miscarriage**, after: ruta.
— **nausea**, with: ant-cr.
— **pains**, with the: dulc. verat.
— **sleep**, during: ars. cact. cina. cup-ac. gels. mur-ac. sant.
— — amel., after: bell. cact.
— — on falling: *bell.* *bry.* cact. calc. camph. chin. *gels.* guai. ign. merc. phos. ph-ac. *spong.* sulph.
— — on awaking: aur. bry. carb-v. colch. dulc. lobel. merc. nat-c. par. sep. stram.
— — on being aroused: phos. secale.
— **sleepiness**, with: acon. arn. *bry.* calc-p. coloc. *puls.*
— **sleeplessness**, with: nx-m.
— **stupor**, with: bapt. lyc. nx-m.
— **sweat**, amel.: æth.
— **trembling**, with: valer.

**Delusions**, fancies, hallucinations, illusions, etc. (Compare with Delirium.)
— **abdomen**, is fallen in, his stomach devoured, his scrotum swollen: sabad.
— **absurd**, ludicrous: cann-i.
— — figures are present: ambr. arg. camph. cann-i. caus. cic. op. tarent.
— **abundance**, has an: *sulph.*
— **active**: hyos.
— **affection** of friends, has lost: aur. hura.
— **angels**, of seeing: ether.
— **animals**, of: absin. æth. ars. *bell.* calc. cham. colch. *hyos.* lac-can. op. puls. sant. stram. sulph. valer.
— — abdomen, are in: thu.
— — black on walls and furniture, sees: bell.
— — bed, on: colch. plb. valer.
— — — dancing on the: con.
— — beetles, worms, etc.: ars. bell. stram.
— — creeping of: lac-can.
— — — in her: stram.
— — cup, in a, moving: hyos.
— — dark colored: bell.

— — dogs, cats, etc.: æth. stram.
— — fire, in the: bell.
— — passing before her: thu.
— — persons are: hyos. stram.
— — jumping at her: merc.
— — rats, mice, insects, etc.: bell. cimic. stram.
— — start out of the ground: stram.
— — unclean: bell.
— — surrounded by wolves: bell.
— **annihilation**, about to sink into: cann-i. carbn-h.
— **ants**, bed seems full of: plb.
— **apoplexy**, thought he would have: arg.
— **argument**, making an eloquent: cann-i.
— **arms** reach the clouds, when going to sleep: pic-ac.
— **arrested**, is about to be: bell. k-bro. plb.
— **assaulted**, is going to be: tarent.
— **babies**, are two in bed: petr.
— **beautiful**: bell. cann-i. coca. sulph.
— — landscape: coff.
— — all things seem, after micturition: erig.
— — even rags seem: sulph.
— **bed**, feels as if not lying on, on waking, 4 A. M.: hyper.
— — — evening, as if some one would get into and no room in it, or as if some one had sold it: nx-v.
— — — as if some one was in, with him: anac. apis. *bapt.* carb-v. nx-v. op. petr. *puls.* rhus. valer.
— — were raised: canth.
— — some one over it: calc.
— — — — under it: am-m. bell. calc. canth. colch.
— — — — stands at the foot, menacing: chloral.
— — — — naked man is wrapped in the bed-clothes with her: *puls.*
— — — — drives him out: rhus.
— — — — tries to take away the bed-clothes: bell.
— **bees**, sees: puls.
— **bells**, hears ringing of: cann-i. ph-ac. thea.
— — — numberless sweet toned: cann-i.
— — — his funeral: ether.
— **bewitched**, thinks he is: cann-i.
— **birds**, sees: bell.
— **blind**, that is: mosch. verat.
— **blood** does not circulate: atrop. sulph.
— — rushed through like roar of many waters: cann-i.
— **body** is brittle: thu.
— — adherent to woolen sack, night, while half awake: cocc-c.
— — black, as if it were: sulph.
— — covered the whole earth: cann-i.
— — delicate: thu.
— — greatness of, as to: *plat.* staph.
— — lighter than air: *op.*
— — had come in pieces and could not get the fragments properly adjusted: phos.
— — scattered about bed, tossed about to get the pieces: *bapt.*
— — shrunken, like the dead: sabad.
— — spotted brown, as if: bell.
— — sweets, is made of: merc.
— — thin, is: thu.
— — three-fold, has a: ars.
— — will putrefy: bell.
— **born**, feels as if newly: cori-r.
— **brain**, has softening: abrot. arg-n.
— **brother** fell overboard in her sight: k-bro.
— **building** stones, appearance of: thu.
— **business**, fancies is doing: bell. bry. canth. cupr. *phos.*
— — thought they were pursuing ordinary: bell.
— **butterflies**, of: cann-i. bell.
— **calls**, some one: anac. ant-c. cann-i. dros. k-ca. *plb.* thu.
— — for absent persons: hyos.
— **cancer**, has a: verat.
— **castles** and palaces, sees: plb.
— **cats**, sees: absin. arn. daph. hyos. puls. stram.
— **caught**, as if he would be: bell.
— **chairs**, thinks he is repairing old: cup-ac.
— **changing** suddenly: cann-i.
— **child**, thinks he is again a: cic.
— — is with companions of his youth: ether.
— — wishes to drive children out of house: fluor-ac.
— — has childish fantasies: lyc.
— **choir**, on hearing music thinks is in a cathedral: cann-i.
— **choked**, thinks he is about to be, night on waking: cann-i.
— — by icy cold hands: canth.
— **Christ**, thinks himself to be: cann-i.
— **churchyard**, visits a: anac. arn. *bell.* stram.

— **clock,** hears strike: ph-ac.
— **clothes,** thinks beautiful: æth. *sulph.*
— — — is clad in rags: cann-i.
— — — would fly away and become stars, on undressing: cann-i.
— **clouds,** strange, settled upon patients, or danced about the sun: cann-i.
— — before the fancy: hepar. mag-m. rhus.
— — heavy black enveloped her: cimic.
— — clouds and rocks above her: mag-m.
— **cockroaches,** swarmed about room: bell
— **companions** seemed half men, half plants: cann-i.
— **confusion,** imagines others will observe her: *calc.*
— **conspiracies** against her father, thought the landlord's bills were: k-bro.
— — against him, there were: ars. plb.
— **cowards,** thinks persons leaving him to be: cann-i.
— **crabs,** of: hyos.
— **crazy,** about to become and no one will take care of him: lil-t.
— — declares she will go: cimic.
— **creative** power, has: cann-i.
— **criminals,** has hallucinations of: alum. am-c. *ars.* bell. carb-v. caus. cina. *cocc.* coff. dig. ferr. graph. *hyos.* nat-c. nit-ac. nx-v. puls. ruta. sil. stront. sulph. verat.
— — thinks has committed a crime: merc.
— — compare Anxiety as if a.
— **cucumbers,** sees on the bed: *bell.*
— **cut** through, as if he were: *stram.*
— **cylinder,** seemed to be a: cann-i.
— **cyphers,** sees: ph-ac. sulph.
— **danger,** impression of: fluor-ac. k-bro. *stram.* valer.
— — to his life: plb.
— — from his family: k-bro.
— **dead** persons, sees: alum. am-c. *anac.* arn. *ars.* aur. bar. *bell.* brom. bry. calc. canth. caus. cocc. con. fluor-ac. graph. hepar. hura. iod. *k-ca.* laur. *mag-c.* mag-m. nat-c. nat-m. nit-ac. nx-v. op. *phos.* *ph-ac.* plat. ran-sc. sars. sil. sulph. sul-ac. thu. verb. znc.
— — morning, on waking, frightened by images of: hepar.
— — midnight, on waking: cann-i.
— — corpse on a bier: cann-i.
— — — of dead brother and child: con.
— — — of sister: agar.
— — — of absent acquaintance on sofa, and has dread: ars.
— — — of tall, yellow corpse trying to share bed with him and promptly ejected: bell.
— — that her child was dead: k-bro.
— — that he himself was dead: apis. camph. lach.
— **debate,** of being in: hyos.
— **delirium** tremens, visions as in: bell.
— **delirious** at night, expected to become: bry.
— — imagines he was delirious: cann-i.
— **deserted,** forsaken, thinks to be: camph. cann-i. carb-an. hydrph. hura. hyos. lil-t. nat-c. *plat.* *stram.*
— **despised,** that is: arg-n. lac-can.
— **devils,** sees: ambr. ars. *bell.* cann-i. cupr. dulc. hyos. k-ca. lach. nat-ca. op. *plat.* puls. stram. sulph.
— — are present: cann-i. op. phos.
— — that he is a demon: camph. cann-i.
— — that persons are: plat.
— **devoured,** had been, by animals: *hyos.*
— **die,** thought he was about to: *acon.* arg-n. cann-i. nit-ac. nx-v. rhus. stram.
— — would, and soon be dissected: cann-i.
— — while walking, thinks he will have a fit or die, which makes him walk faster: *arg-n.*
— **diminished:** cann-i. cinnam. grat. lac-can. sabad. sulph.
— — everything in room is, while she is tall and elevated: plat.
— — left side of body is smaller: cinnam.
— — whole body is: agar.
— — abdomen has fallen in: sabad.
— — short: lac-can.
— — shrunken: sabad.
— — — fingers and toes are: cann-i.
— — small: grat.
— — thin, is too: thu.
— **dirty,** that he is: rhus. lac-can.
— — eating dirt: verat.
— **disabled,** that she is: cit-v.

— **disgraced**, will be: sabin.
[— **disease**, has incurable: arg-n. cact. plb.
— — is deaf, dumb, and has cancer: verat.
— — has an unrecognized disease: raph.
— **divided**, into two parts: *bapt.* cann-i. petr. puls. sil. stram. thu.
— — — — or cut in two: plat.
— — — — — and could not tell of which part he had possession, on waking: thu.
— **divine**, thinks is: cann-i. stram.
— **doctors**, thought three were coming: sep.
— **dogs**, sees: arn. bell. calc. lyc. merc. puls. sil. stram. sulph. verat. znc.
— — attack him: stram.
— — biting his chest: stram.
— — black: bell.
— — swarm about him: bell. stram.
— **dolls**, people appeared like: plb.
— **double**, of being: anac. bapt. cann-i. glon. lil-t. mosch. petr. stram.
— —See also Divided.
— — sensations present themselves in a double form: cann-i.
— **dragged** from the lowest abyss of darkness, on waking: thea.
— **dreaming** when awake, imagines himself: bell.
— **drinking**: bell.
— **driving** sheep: acon.
— — peacocks: hyos.
— **dumb**, thinks is: verat.
— **eaten**, as if would be: stram.
— **elevated**, in air: nit-ox.
— — carried to an elevation: œna.
— — bed were raised: canth.
— **emaciation**: sil.
— **emperor**, thought himself an: cann-i.
— — talked of: carbn-s.
— **enemy**, lies in wait: alcoh.
— — is under the bed: am-m.
— — every one is, an: merc.
— — pursued by: dros. *lach.*
— — surrounded by: anac. carbn-s.
— **engaged** in some occupation, is: acon. ars. atrop. bell. cann-i. cupr. cupr-ac. ether. hyos. hydrph. plb. rhus. stram. verat.
— — in ordinary occupation: ars. atrop. bell. ether. plb. stram.
— **enlarged**: acon. alum. bell. *cann-i.* glon. nat-c. nx-v. op. *plat.* sabad. stram. znc.
— — parts of body: alum. op.
— — head is: acon. cann-i. znc.
— — eyes are: bell. op.
— — eye-lashes are: cann-i.
— — chin is: glon.
— — scrotum is swollen: sabad.
— — one leg is longer: cann-i.
— — is very tall: op. pallad. plat. stram.
— — persons are: cann-i. caus.
— — distances are: cann-i. nx-m.
— — objects are: cann-i.
— **entering**, fancies some is, night: con.
— **epilepsy**, fancies he has: atrop.
— **erroneous**: bell. canth. hyos. nx-v. stram. verat.
— **eternity**, that he has lived an: ether.
— — that he was in: cann-i.
— **excited**: coff.
— **executioner**, visions of an: stram.
— **existence**, doubt if anything had: agn.
— — doubted his own: cann-i.
— **expanding**, thought passers-by were: cann-i.
— — See also Enlarged.
— **experienced** before, thought everything had been: k-bi.
— **eyes**, falling out: crot-c.
— — are larger: op.
— — lashes are longer: cann-i.
— **faces**, sees: ambr. arg-n. calc. cann-i. caus. op. sulph. tarent.
— — wherever he turns his eyes, or looking out from corners: phos.
— — diabolical, crowd upon him: ambr.
— — of distinguished people: cann-i.
— distorted, day, on lying down: arg-n.
— — on closing the eyes: ars. bell. calc. carb-v. caus. chin. samb.
— — ridiculous: cann-i.
— — ugly faces seem pleasing: cann-i.
— **fail**, everything will: arg-n.
— **fall**, as if things would: hyos.
— **family**, does not belong to her own: plat.
— **fanciful**: lach. *stram.*
— — in slumber: ant-c.
— **fault-finding**: bar-ac. plb. rhus.
— **fight**, sees a: op.
— **figures**, sees: cocc. nx-m. plb. sant. spong.
— — See Spectres.

— — large black about to spring on him: mosch.
— — gigantic: atrop.
— — marching in the air, evening, while half asleep: nat-c.
— — hurled bottle at: chloral.
— **fingers** cut off: mosch.
— — finger-nails seem as large as plates, during drowsiness: cann-i.
— **fire,** visions of: alum. anac. ant-t. ars. *bell.* *calc.* calc-p. clem. creos. croc. daph. hepar. laur. mag-m. nat-m. nitr. phos. plat. puls. rhod. rhus. spig. spong. stann. sulph. znc.
— — distant home on: bell.
— — head is surrounded by: am-m.
— — house on: bell. hepar. stram.
— — neighbor's house on, morning, waking, in a fright: hepar.
— — room is on: stram.
— — world is on: hepar.
— **fishes,** flies, etc.: bell.
— **floating** in air: cann-i. canth. hura. k-bro. nx-m.
— — evening: bell.
— — on closing eyes: penth.
— — when walking: asar.
— — bed is suspended: bell. stram.
— — is not resting in bed: sticta.
— — leg is floating: sticta.
— **fluid,** ethereal, surrounded by, resisting passage: cann-i.
— **flying,** sensation of: asar. camph. cann-i. ether. œna. op.
— — from a rock into dark abyss, on going to bed: cann-i.
— **footsteps,** behind him: crot-c.
— — in next room: nat-p.
— **forsaken.** See Deserted.
— **foul,** everything appears: curare.
— **fowls,** sees: stram.
— **friend,** thinks she is about to lose a: hura.
— — has lost the affection of: aur.
— — has offended: ars.
— **frightful**: absin. atrop. bell. camph. coca. nicot. op. rhod. stram.
— — evening: calc.
— — night: camph. carb-v. nit-ac. phos. tabac.
— — on closing eyes: calc. caus. lach.
— — on falling asleep: chin.
— — of past events: spong.
— **furniture,** imagines it to be persons, night on waking: nat-p.
— **gallows,** visions of, with fear of: bell.
— — **geese,** threw themselves into water, thinking themselves to be: con.
— — sees: hyos.
— **ghosts.** See Spectres.
— **giants,** sees: bell.
— **giraffe,** imagines himself to be a: cann-i.
— **God,** sees: ether.
— — is the object of God's vengeance: ether. k-bro.
— **goitre,** imagines he has a: indg.
— — has one which he cannot see over when looking down: znc.
— **grandeur,** magnificent visions of: carbn-s. coff.
— **grasp,** has fantasies beyond his: viol-od.
— **great** person, is: æth. bell. *cann-i.* cupr. hydrph. phos. *plat.* sulph. verat.
— **groans,** fancies he hears: crotal.
— — **growling,** as of a bear: mag-m.
— **hall,** illusion of a gigantic: cann-i.
— **hanging,** sees persons: ars.
— — three feet from the ground, on falling asleep: hura.
— — on standing high, seems as if: phos.
— **hand,** midnight visions of something taking her: canth.
— — visions of white, outspread, coming toward face in the darkness: benzin.
— **harlequin,** as if he were a: hyos.
— **hat** is a pair of trousers, which he tries to put on: stram.
— **head** heavy, his own seemed too: bry.
— — large, seems too: acon.
— — large heads, make grimaces, evening on closing eyes: euphr.
— — **pendulum,** seems an inverted, oscillating: cann-i.
— — of deceased acquaintances, without bodies, at night: nx-v.
— — monstrous, on distant wall of room: cann-i.
— — thinks disease will break out of: stram.
— — transparent and speckled brown: bell.
— — shaking the: bell. cham.
— **hear,** thinks he cannot: mosch.
— — — he is deaf and dumb: verat.
— — a bell: ph-ac.
— — footsteps: canth. carb-v.

— **heat,** has a furious, radiating from epigastrium: cann-i.
— **heaven**, is in: cann-i. op.
— **heavy**, is: nat-c. thu.
— **hell**, is in: camph. cann-i. merc.
— — at gate of, obliged to confess his sins: agar.
— — in shadows of, midnight, on waking: cann-i.
— — suffers the torments of without being able to explain: merc.
— **help**, calling for: plat.
— **herbs**, gathering: bell. cupr.
— **hide**, tries to: bell. puls.
— **hogs**, mistakes men for: hyos.
— **hole**, small, appears like a frightful chasm: agar.
— **home**, thinks is at, when not: cann-i. hyos.
— — everything at, has changed: arg-n.
— — thinks is away from: acon. coff. op. valer. verat.
— — wants to go: bell. bry. lach. op. verat.
— **horses**, sees: bell. mag-m. znc.
— — is on horseback: cann-i.
— **house** is full of people: ars. cann-i. con. lach. lyc. merc. nat-m. nx-v. sil. stram.
— — seems movable: cann-i.
— — not in right place, while walking, after headache: glon.
— — on each side would approach and crush him, with vertigo: arg-n.
— — is surrounded: stram.
— **humility** and lowness of others, while he is great: plat. staph.
— **hunter**, thinks is a: cann-i. verat.
— **identity**, doubted his own: phos. plb.
— **images**, phantoms, sees: ambr. arg-n. ars. *bell.* calc. camph. carb-an. carb-v. caus. cham. cic. coca. dros. dulc. hell. hepar. hyos. k-ca. lach. merc. nat-c. nit-ac. nx-v. *op.* phos. ph-ac. plat. puls. rhus. sep. *stram.* sulph. verat.
— — black: arn. ars. *bell.* op. plat. puls.
— — frightful: ars. bar. *bell.* calc. carb-v. caus. chin. coca. con. croc. graph. hepar. hyos. k-ca. laur. lyc. mang. merc. mur-ac. nat-c. nit-ac. nx-v. *op.* petr. phos. ph-ac. puls. rhod. rhus. sars. sil. spong. *stram.* sulph.
— — rising out of the earth: stram.
— — sees all over: merc. *sil.*
— — side, at his: stram.
— — ever changing: carbn-ox.
— — fantastic, when alone: fluor-ac.
— — evening: lyc.
— — before going to sleep: carb-an.
— — on closing the eyes, in bed: sulph.
— — wall, on the: samb.
— — — hateful, afternoon, during sleep: lyc.
— — See Faces, Figures, Men, Spectres, Visions, etc.
— **immovable**, sitting: cham. hyos. puls. stram.
— **inanimate** objects are persons: bell. nat-p.
— **inferior**, on entering house after a walk, people seem mentally and physically: plat.
— **influence**, has a powerful: cer-b.
— **injury**, is about to receive: ars. cann-i. con. lach. lyc. merc. nx-v. sil. stram. sulph.
— — is being injured: bry. cact. canth. elaps. hydrph. k-bro. phos. rhus. stram. sulph.
— — his fingers and toes are being cut off: mosch.
— **inkstand**, fancied he saw one on bed: lact.
— — — he was one: cann-i.
— **iodine**, illusions of fumes of: iod.
— **insects**, sees: *ars.* bell. phos. puls. *stram.*
— — shining: bell.
— **insulted**, thinks he is: alcoh. bell. k-bro. tarent.
— **island**, is on a distant: phos.
— **journey**, as if on a: *bell.* brom. cann-i. crotal. hyos. lach. mag-m. nat-c. op. sang. sil.
— **jumped** up on the ground before her, all sorts of things: brom.
— **knowledge**, thought he possessed infinite: cann-i.
— **labor**, pretends to be in, or thinks she has pains: verat.
— **lady**, fancied herself a noble: phos.
— **large**, people seemed too, during vertigo: caus.
— — parts of body seem too: alum. op.
— — self seemed too: stram.
— — — — on entering the house: plat.
— — See Enlarged.
— **laughed** at, imagines she is: bar-ac.
— **leg** is too long: cann-i.
— — is a tin case filled with stair-rods: cann-i.

— **lies** crosswise: stram.
— **life,** symbols of, all past events, revolve rapidly on wheels: cann-i.
— — is threatened: k-bro.
— **light,** on falling asleep thinks there is too much in room: ambr.
— **limb,** one is double: petr.
— **living** under ordinary relations, thinks is not: cic.
— **locomotive,** imagines himself to be: cann-i.
— **longer,** things seem: berb. camph. creos. dros. nit-ac. sulph.
— **lost,** fancies herself: aur. hura.
— — — on waking: æsc-h.
— **lunatic,** as if a: anac. hyos.
— **lying** near him, fancies some one is: petr.
— — crosswise: stram.
— — See also Bed.
— **ludicrous**: cann-i.
— **machine,** thinks he is a working: plb.
— **maelstrom,** seemed carried down a psychical: cann-i.
— **man,** who hung himself, saw: ars.
— — in the room intending to perforate his throat with a gimlet: merc-i-fl.
— — muffled, starts from the wall, when walking: cann-i.
— — thinks men are on the bed, at night: merc.
— — naked man in bed: *puls.*
— — old men with long beards and distorted faces, sees: laur.
— **mandarin,** mistook friends for a Chinese: cann-i.
— **marble** statue, felt as if he were: cann-i.
— **marriage,** is going to be: hyos.
— — must dissolve: fluor-ac.
— **masks,** sees: k-ca. *op.*
— **melancholy**: alum.
— — while half awake, at night: nx-v.
— — thoughts of himself or a friend lying on a bier: anac.
— **melting** away, sensation of, worse from change, better in recumbent position: sumb.
— **mice,** sees: bell. *calc.* colch. mag-s. op.
— **mind** and body separated: anac.
— **misfortune,** inconsolable over fancied: *verat.*
— **money,** as if counting: alum. bell. cyc. mag-c. znc.
— — is sewed up in clothing: k-bro.
— — talks of: carbn-s.
— **monsters,** visions of horrible: bell. camph. cann-i. cic. lac-can. samb. stram. tarent.
— — on falling asleep and on waking: ign.
— — See also Spectres.
— **mountain,** thought himself to be on the ridge of a: cann-i.
— **mouth,** fancied living things were creeping in: merc.
— **moving,** as if things were: phos.
— **murdered,** sees some one: calc.
— — as if he would be: am-m. bell. hyos. ign. k-ca. lact. lyc. mag-c. merc. op. phos. stram. verat. znc.
— — thinks persons were bribed to murder him: cann-i.
— — are conspiring to murder him: plb.
— — that he was killed, roasted, and eaten: stram.
— — that every one around him is a murderer: plb.
— — thought her mother had been: nx-v.
— **mushroom,** fancies he is commanded to fall on his knees and confess his sins and rip up his bowels, by a: agar.
— **music,** fancies he hears: cann-i. croc. stram. thu.
— — delightful: plb.
— — evening, hears the music heard in the day: lyc.
— — sweetest and sublimest melody: cann-i.
— — unearthly: ether.
— **mutilated** bodies, sees: ant-c. arn. con. mag-m. merc. *nx-v.* sep.
— **mystery,** everything around seemed a terrifying: cann-i.
— — mystic hallucinations: ether.
— **naked,** thinks is: stram.
— **narrow,** everything seems too: plat.
— **needles,** sees: merc. *sil.*
— **neglected** or despised, is: arg-n.
— **new,** everything is: stram.
— **newspapers,** thinks he sees: atrop.
— **noise,** hears: bell. calc. canth. carb-v. cham. colch. con. mag-m.
— — — clattering about bed: calc.
— — — of shouts, vehicles: cann-i.
— — — steps in room: canth.
— — — knocking under bed: canth.
— **nose,** has a transparent: bell.

— — has some one else's: lac-can.
— — takes people by: merc.
— **numeral**, appeared nine inches long, night on waking, better on other side: sulph.
— **nursing** her child, imagines she was: atrop.
— **nuts**, as if cracking: hyos.
— **objects**, brilliantly colored: bell.
— — crooked: glon.
— — appear different: nat-m.
— — flight from: stram.
— — glittering: bell.
— — numerous, too, in room: phys.
— — sometimes thick, sometimes thin, on closing eyes: camph.
— — tries to seize: ars. atrop. bell. hyos. œna.
— — are present: cann-i.
— — in open air: atrop.
— **obscene**: stram.
— **officer**, thinks he is an: agar. bell. cann-i. cupr-ac.
— **ox**, as if riding on: bell.
— **paradise**, thought he saw: coff.
— — is in: cann-i. op.
— **past**, of events, long: atrop.
— **peacocks**, as if chasing: hyos.
— **people**, persons, are looking at him: rhus.
— — sees: bell. hyos. op. mag-c. *puls.* rheum. sep. stram.
— — some one is behind him: anac. brom. casc. crotal. staph.
— — is beside him: anac. apis. ars. bell. camph. carb-v. nx-v. petr. valer.
— — — — — — doing as he does: ars.
— — See Bed, Room, etc.
— **persecution**, fancies himself to be subjected to: china. hyos. k-bro. stram.
— **pieces**. See under Body, Divided, etc.
— **places**, of being in different: cann-i. lyc. plb. raph.
— — at night on waking thought to find himself in strange and solitary: par.
— — in wrong: hyos.
— **pleasing**: atrop. cann-i. op. stram.
— — morning, after sleep: bell.
— **poisoned**, thought he had been: hyos. plat-m.
— — that he was about to be: plb. rhus.
— — medicine being: lach.
— — See under Fear of.
— **policeman** come into house, thought he saw: hyos. k-bro.
— **poor**, thinks he is: bell. calc-fl. hepar. nx-v. *sep.* stram. valer.
— — family will starve: ars.
— **possessed**, as if: anac. bell. hyos.
— **pins**, hunts: sil.
— — is afraid of: spig.
— **pregnant**, thought herself: ign. verat.
— **present**, some is: hyos. lyc. thu.
— — See People.
— **prince**, is a: verat.
— **proud**: plat. stram. verat.
— **pulling** one's hair: bell.
— **pursued**, thought he was: absin. anac. bell. plb.
— — by enemies: absin. lach.
— — by friends: plb.
— — by ghosts: lepi.
— — by police: alcoh. k-bro.
— — by murderers, robbers: alcoh.
— — by soldiers: absin.
— — See also Persecuted.
— **rabbits**, sees: stram.
— **railway** train, thought he was obliged to go off by: atrop.
— **rain**, from having wet cloth on head, thought he had been out in: atrop.
— **rats**, sees: bell.
— — of all colors: absin.
— — running across room: æth.
— **reading** behind him, some is, which makes him read the faster: mag-m.
— **reason**, is losing: acon. calc. cann-i. chel. lam. merc. nat-m. tanac.
— — worse walking in open air: acon.
— **reproaches**, is neglected and deserves: aur.
— **repudiated** by relatives, thinks is: hura.
— **rich**, felt himself unusually: alcoh. bell. cann-i. sulph.
— **riding** on an ox: bell.
— — — a horse: cann-i.
— **roasted**, as if would be: stram.
— **robbing**, of: k-bro.
— **room**, is in another: cann-i.
— — is a garden: calc.
— — sees people in, at bedside: atrop.
— — — on entering: lyc.
— — is like the foam of a troubled sea: secale.
— — walls of, seemed gliding together: cann-i.
— — — sees horrible things on the walls: bell. cann-i. samb.

— **saw**, thought he was a huge, darting up and down: cann-i.
— **scorpions**, sees: op.
— **scratching** on linen or similar substance, thought some one was: asar.
— **scrotum**, thinks his is swollen: sabad.
— **seized**, as if: canth.
— **sensations**, misrepresents his: bell.
— **serpent**, a crimson, fastening on his neck: bell.
— **servants**, thinks he must get rid of: fluor-ac.
— **sewing**, imagined herself to be: atrop.
— **ship**, thinks they are on board of, in a storm: alcoh.
— **shoot**, tries to, with a cane: merc.
— **shopping**, with her sister: atrop.
— **shouting**, fancied himself to be: cann-i.
— **sick**, imagines himself to be: bell. calc. sabad.
— — that he was going to be: nat-c.
— — that two sick people were in bed, one of whom got well and other did not: secale.
— **side**, imagined himself alive on one side and buried on the other: stram.
— — that she did not own her left: sil.
— **singing**, fancied himself to be: cann-i.
— **skeletons**, sees: op.
— **sleeping**, while awake insists that he was: acon. alcoh.
— **small**, things appear: *plat.* stram.
— — sensation of being smaller: acon. tarent.
— — See also Diminished.
— — things grow smaller: camph. carb-v. nit-ac.
— **snakes** in and around her: lac-can.
— **soda-water**, thinks he is a bottle of: cann-i.
— **sold**, as if would be: hyos.
— **soldiers**, sees: *bell.* bry. nat-c. op.
— — on his bed: lact.
— — cutting him down, better on getting cool: bry.
— — march silently past: cann-i.
— **soot**, showers of fell on him: cann-i.
— **sorrow**, thinks every one he meets has a secret: cann-i.
— **soul**, fancied body was too small for, or that it was separated from: cann-i.
— **sounds**, listens to imaginary: hyos.
— **space**, fancied he was carried into, when lying: coca.
— — expansion of: cann-i.
— **spectres**, ghosts, phantoms, etc., sees: alum. ambr. am-c. ant-t. *ars.* atrop. *bell.* bov. carb-v. cocc. dulc. ign. k-ca. lach. merc. nat-c. nat-m. nit-ac. op. phys. plat. puls. sars. sep. sil. spig. stram. sulph. tarent.
— — on closing eyes: arg-n. bell. bry. calc. chin. ign. **led.** nat-m. samb. stram. thu.
— — in fire: bell.
— — black forms, when dreaming: arn. ars. puls.
— — clutches at: hyos.
— — of death as a gigantic black skeleton: crot-c.
— — which continues to enlarge until it disappears, morning on waking: dulc.
— — feel as if spectre would appear in evening: brom.
— — is pursued by: stram.
— **speeches**, makes: arn. cham. lach.
— **sphere**, thought himself a: cann-i.
— **spinal** column a barometer: cann-i.
— **spinning**, is: hyos. stram.
— — dreams he is: sars.
— **spotted** brown, is: bell.
— **spittle**, licking up: merc.
— **square**, fancies of a colossal surrounded by houses a hundred stories high: cann-i.
— **stamps** with the feet: ant-c. verat.
— **stars**, saw in his plate: cann-i.
— **starve**, family will: ars.
— — See also Poor.
— **stomach**, thinks has corrosion of: ign. sabad.
— **stones**, taking into mouth: merc.
— **stove** for a tree, mistakes: hyos.
— **strange**, everything is: cic. nx-m. plat. staph. stram.
— — familiar things seem: atrop. bar-m. bell. calc. cann-i. cic. croc. glon. hydrph. hyos. mag-m. mosch. op. plat. rhus. stram. verat.
— — — — are horrible: plat.
— — — — are ludicrous: cann-i. hyos. nx-m.
— — — places seemed: rhus-r.
— — — — — after headache: glon.
— — land, as if in a: bry. par. plat. verat.
— — strangers seemed to be in the room: tarent.

— — — about her while knitting: mag-s.
— — — looking over shoulder: brom.
— — — friends appear as: stram.
— **superhuman**, is: cann-i.
— — is under control: lach.
— **surroundings** are capacious: ferr.
— surrounded by friends, is: bell. cann-i.
— **swimming**, is: cann-i.
— **swollen**, is: cann-i. carb-s.
— — See also Enlarged.
— sword, hanging over head: am-m.
— **talking**, imagines he hears: elaps. stram.
— — fancies herself: raph.
— — irrationally: nit-ac.
— — loudly and incoherently, to imaginary persons: bell.
— — of persons as though near, about midnight: sep.
— — of with dead people: *bell.* nat-m. stram.
— — to inanimate objects: stram.
— **tall**, fancies herself very: stram.
— — had grown, while walking: pallad.
— — as if he were: stram.
— — things grow taller: camph. creos. dros. nit-ac. sulph.
— **thieves**, sees: alum. ars. aur. bell. k-ca. mag-c. mag-m. merc. nat-c. nat-m. petr. phos. sil. verat. znc.
— — in house: cann-i. merc. nat-m. sil.
— — after a dream, and will not believe the contrary until search is made: nat-m.
— — dreams of robbers, is frightened on awaking, and thinks dream is true: verat.
— — that house and space under bed are full of: ars.
— **thin**, is getting: sulph.
— — body is: thu.
— **throat**, some one with ice-cold hands took her by the: canth.
— **time**, exaggeration of duration of: cann-i.
— — seems earlier: sulph.
— — — too short: cocc. coca. thea. therid. thu.
— — — too long: cann-i. nx-m.
— — See Ennui and Time.
— **toes**, cut off: mosch.
— **tongue**, seems to reach the clouds, when going to sleep: pic-ac.
— — pulling out: bell.
— **tormented**, thinks is: *chin.*
— **touching** everything: bell.
— **toys**, fancied himself playing with: atrop.
— — object seemed as attractive as: cic.
— **transferred** to another room: coloc.
— — to another world: cann-i.
— **transparent**, seemed to be: cann-i.
— — head and nose are: bell.
— **traveling**, of: cann-i.
— — through worlds: ether.
— **trees**, seem to be people in fantastic costume, afternoon, while riding: bell.
— **trembling**, of everything on him, at night, when only half awake: sulph.
— **troubles**, broods over imaginary: naja.
— **turtles**, sees large, in room: bell.
— **typhoid** fever, thought he would have: naja.
— **unpleasant**: bell.
— — distinct from surrounding objects: bell.
— **unreal**, everything seems: cann-i. lil-t. staph.
— **unseen**, thing: tarent.
— **vagina**, living things creep into, at night: merc.
— **vanish**, seems as if everything would: lyc.
— **vegetable** existence, thinks he is leading a: cann-i.
— — thinks he is selling green: cup-ac.
— **vengeance**, thinks she is singled out for Divine: k-bro.
— **vermin**, sees crawl about: alum. am-c. ars. bov. k-ca. mur-ac. *nx-v.* phos. ran-sc. sil.
— — his bed is covered with: ars.
— **vexations** and offenses, of: cham. chin. dros.
— **violence**, illusions about: k-bro.
— **visions**: alum. ambr. bell. calc. cham. graph. hyos. lyc. nat-m. nx-m. op. plat. rhod. sep. sil. spong. stram. sulph.
— — See also under Faces, Images, Spectres, etc.
— — imaginary power of: cann-i.
— — fantastic: ars. hyos. op.
— — beautiful: bell. coca.
— — horrible: bell. merc. op. stram.
— **vivid**: cham. ether. op. spong. *stram.*

— **voices,** hears: anac. cann-i. *cham.* lyc. manc. plb. stram.
— — confused, worse swallowing or walking in open air: benz-ac.
— — distant: bell.
— — in bed, worse when listening intently: abrot.
— — are in his abdomen: thu.
— — his own sounds strange and seems to reverberate like thunder: cann-i.
— **walk,** fancies he cannot: ign.
— — — that he walks on his knees: bar.
— **walls,** is surrounded by high: cann-i.
— **want,** fancied that he would come to: calc-f.
— — that they had come to: cann-i.
— — See Poor.
— **warts,** thinks has: mezer.
— **washing,** of: bell.
— **water,** illusion of blue: cann-i.
— — of disasters by: cann-i.
— — a spoonful, seems like a lake: agar.
— — when drinking thought it delicious nectar: cann-i.
— — sees flowing: merc.
— **wealth** imaginations of: bell. cann-i. k-bro. verat.
— **weight** has no: cann-i. op.
— **well,** thinks he is: cinnb. creos.
— **where** he is, knows not: atrop. bov. merc.
— — on waking: puls.
— — — — from a dream: cann-s.
— **wind** sighing in chimney sounded like the hum of a vast wheel, and reverberated like a peal of thunder on a grand organ: cann-i.
— **wires,** is caught in: cimic.
— **wolves,** of: bell.
— **women,** fancies his mother's house is invaded by lewd: k-bro.
— — are evil and will injure his soul: puls.
— — illusions of old and wrinkled: cann-i.
— **work,** is hard at: rhus.
— — is hindered at: chin.
— **worms,** imagined vomitus to be a bunch of: cann-i.
— **wretched,** thinks she looks, when looking in a mirror: nat-m.
— **wrong,** fancies he has done: dig. merc.
— — has suffered: lach. naja.

**Deserted.** See Abandoned, Forsaken, etc.
**Desires.** See the things desired.
**Despair:** *acon.* agar. agn. all-c. aloe. alum. *ambr.* am-c. ant-t. arn. ARS. aster. *aur.* bad. bov. brom. bry. *calc.* cann-i. canth. carb-an. carb-v. *caus.* cham. chel. chin. cocc. colch. cupr. der. dig. eup-per. gamb. *graph.* hell. helon. hepar. hura. hydr-ac. hyos. *ign.* iod. k-bi. k-bro. *lach.* lyc. merc. morph. naja. nat-c. *nat-m.* nat-s. nitr. nit-ac. nx-v. orig. petr. plb. *psor. puls.* rhus. secale. sil. stann. stram. *sulph.* therid. valer. *verat.*
— about others: *aur.*
— of **health**: calc. staph.
— — recovery: *acon.* ars. bapt. bar. bry. calc. cann-i. cham. creos. hell. hura. ign. k-ca. nat-s. nx-v. sil. therid.
— — — despairs of recovery during convalescence: *psor.*
— in **fever,** intermittent, during the chill: *acon.* ant-t. ARS. *aur.* bry. *calc.* *cham.* cupr. graph. hepar. IGN. merc. nx-v. rhus. *sep.* verat.
— — — during the hot stage: *acon.* ars. *carb-v.* cham. con. graph. ign. puls. sep. *spong.* stann. sulph. verat.
— — — during the sweat: ARS. calc. *carb-v. cham. graph.* lyc. *sep.* stann. verat.
— **pains,** with the: *ars. aur.* carb-v. cham. colch. lit-t. nx-v.
— — from pain in stomach: ant-cr. *coff.*
— **religious** (of salvation, etc.): ars. *aur.* calc. lach. lit-t. *lyc.* psor. puls. stram. *sulph. verat.*
— **social** position, of: verat.
— **vomiting,** when: ars-h.
**Despises.** See Contempt.
**Despondency,** hopelessness, etc.: acon. æsc-h. agn. aloe. ambr. am-m. anac. ant-t. arg-n. *arn. ars. aur.* bapt. bar. bell. *bry.* calc. camph. canth. carb-an. carb-v. *caus.* cham. chin. cocc. coff. colch. con. creos. dig. erig. gad. gels. GRAPH. ham. hell. hepar. hura. hydr. hyos. *ign.* iod. k-bi. k-ca. *lach. laur.* lil-t. lyc. mag-m. merc. MEZER. murx. mur-ac. nat-ars. nat-c. nat-m. nat-p. nicc. nit-ac. *nx-v.* op. petr. phos. ph-ac. pin-s. plan. plb. *psor. puls.* rhus. *ruta.* sabad. secale. sep. sil. spig. stann. staph. sulph. sul-ac. sumb. tabac. thu. tilia. verat. verb. xanth. znc.

— compare with Anxiety, Despair, Sadness, etc.
— believes all he undertakes will fail: arg-n. lac-can.
— **dinner**, after: tilia.
— **domestic** affairs, about: viol-tr.
— **evening**: *ant-t.* senec. nat-p. znc.
— **fever**, with: eup-per.
— **nausea**, after: tabac.
— **night**, at: plat. *rhus.*
**Destructiveness** of things: bell. cupr. merc-i-fl.
— of clothes: sulph. tarent.
— — — cuts them up: verat.
**Dictatorial** conduct: camph. caus. ferr. lach. lyc. merc.
**Disagreeable.** See Ill-humor.
**Disconcerted**: brom. ign.
**Disconsolate.** See Sad.
**Discontented**, displeased, dissatisfied, etc.: acon. æth. agn. alum. am-c. *am-m.* ang. arn. ars. aur. bell. *bism.* bry. calc. calc-p. canth. carb-an. caps. caus. chin. cic. *cina.* cinnb. clem. cocc. colch. coloc. con. *creos.* croton. cupr. dulc. ferr. ham. hepar. hipp. ign. *indg.* k-ca. laur. *led.* lept. lil-t. lyc. mag-s. mang. men. merc. mur-ac. nat-c. *nit-ac.* nitr. ol-an. orig. par. petr. phos. ph-ac. plat. plb. prun. *puls.* ran-b. rhod. *rhus.* ruta. sep. *sil.* spong. stann. staph. *sulph.* tarent. thu. tilia. viol-tr.
— with himself: *agn.* aloe. ars. aur. bell. bry. calc-p. caus. cham. cinnb. cob. cocc. hepar. k-ca. lyc. mang. men. merc. mezer. mur-ac. nit-ac. ph-ac. puls. ruta. sulph. tarent. viol-tr.
— — is sorry for himself: agar. nit-ac.
— with his surroundings: ang. *cham.* chel. par. men. merc. mezer. plat.
— with everything: am-c. apis. arn. bism. cann-s. cham. coff. colch. eugen. grat. hepar. hipp. ign. iod. nat-c. puls. samb. sep. spong. sulph. thea.
— **air**, in open: mur-ac.
— **morning**: hipp. plb. puls.
— — before stool: *borax.*
— **evening**: fluor-ac. hipp. ign. ran-b. rhus.
— **coition**, after: *calc.*
— **eating**, after: fluor-ac.
— **menses**, during: cast. tarent.
— **pain**, during: hepar.
— **rainy** weather, in: aloe.
— **stool**, before: borax.
— **weeping**, amel.: nit-ac.
**Discouraged**: *acon.* agar. agn. aloe. alum. ambr. *anac.* ang. ant-c. ant-t. arg. arn. *ars.* aur. bar. bell. bry. calad. calc. camph. canth. carb-an. carb-v. caus. cham. *chin.* chin-s. cocc. coff. colch. coloc. con. conv-d. cupr. dig. *dros.* gran. graph. hell. hepar. hydr-ac. hyos. ign. iod. ipec. k-bi. k-ca. *lach.* laur. lyc. merc. merc-c. mur-ac. nat-c. nat-m. nat-s. nitr. nit-ac. nx-v. olnd. op. *petr.* phos. ph-ac. plb. podo. psor. *puls.* ran-b. rhus. sabin. secale. *sep.* sil. spig. stann. stram. *sulph.* sul-ac. tabac. tarent. therid. thu. valer. *verat.* verb. viol-tr. znc.
— after coition: sep.
— **evening**: ferr-p. ran-sc. *rhus.*
— — when eating, amel: tarent.
— when walking: am-c. ph-ac.
**Disgust**: cimx. puls. sulph.
— See also Loathing.
**Disheartened.** See Discouraged.
**Disobedience**: acon. agn. *am-c.* am-m. arn. canth. caps. caus. *chin. dig.* guai. *lyc.* nit-ac. nx-v. phos. spig. sulph. *viol-tr.*
**Despise**, disposition to: ars. ipec.
**Displeased.** See Discontented, Vexed, etc.
**Disputing**: caus. ferr. hyos. lach. merc.
— See also Contradict.
**Dissatisfied.** See Discontent.
**Dissected**, thinks he would be: cann-i.
**Distance**, inaccurate judge of: *cann-i. stram.*
— exaggerated is: *cann-i.* glon.
— compare with Size.
**Distraction**: *acon.* ars. canth. clem. colch. cyc. hyos. k-ca. *lyc.* mag-m. mosch. nat-c. nx-m. ol-an. orig. phos. plect. raph. sil. *spong.* stann. sulph. thu. verat.
— compare with Mind, absence of, etc.
— during conversation: lyc.
— while reading: lach.
— — studying: hell.
— — writing: acon. mag-c.
**Distrustful.** See Suspicious.
**Disturbed**, averse to being: *bry.* gels.
**Dogs**, afraid of: chin. stram.
**Dogmatic.** See Dictatorial.
**Doleful.** See Sad.
**Domineer**, desires to: lyc.
**Doubtful**: ars. aur. bar. chin. graph. mur-ac. nx-v. sil. thu.

— compare with Despair, Fear, Anxiety, etc.
— of recovery: *acon.* agn. bry. calc. ign. k-ca. lach. nx-v. phos. ph-ac. psor. puls. sep. sulph.
— of soul's welfare: ars. aur. bell. croc. dig. hyos. lach. lil-t. nx-v. puls. selen. stram. *verat.*

**Dread.** See Fear.

**Dreaming**, while awake (dreamy mood): acon. anac. ang. arn. ars. bell. cham. cupr. hepar. hyos. lach. lil-t. merc. nat-m. ol-an. op. phos. ph-ac. phys. sil. stram. thu. ziz.
— about the future, poetical: olnd.
— compare Fancies.

**Drinking** on the sly: sulph.

**Drown** himself, disposition to: ant-c. bell. hell. hyos. puls. rhus. secale. verat.

**Drunken**, seems as if. See Stupefaction under Head.

**Dullness.** See under Head.

**Duty**, feels as if had neglected: cyc. nat-ars. puls.

— — — — during headache: naja.

**Earnestness**: æth. am-c. am-m. bov. cham. chin. *cina.* cocc. cyc. euph. ferr. grat. led. nx-m. plat. ph-ac. spig. staph. thu. tilia.
— See also Serious.

**Eccentricity**: verat.

**Ecstacy**: acon. agar. agn. ang. ant-c. arn. bell. bry. cann-i. cham. coff. *lach.* olnd. op. phos. ph-ac. plat. plb. valer.
— amorous: op. pic-ac.
— — during sleep: phos.

**Elated**: coca. iod.
— alternating with sadness: senec.
— compare Cheerful, Contented, etc.

**Elevation**, mental: op.
— morning, on walking in open air: cinnb.

**Embarrassed**: chin. hyos. sulph.
— in company: *ambr.* carb-v.

**Embraces** companions: agar.
— anything, in morning, worse in open air: plat.

**Emotions**, predominated by the intellect: valer. viol-od.
— easily excited: morph. sumb.

**Ennui**: alum. bar. borax. camph. chin. con. elaps. ferr. hura. hydr. lach. lyc. mag-m. manc. mezer. *nat-c.* nitr. *nx-v.* petr. plat. plb. tarent. znc.
— see Loathing of Life.

**Envy**: ars. bry. calc. lach. lil-t. lyc. nat-c. *puls.* sep. staph.

**Escaping**, fleeing from house: acon. *bell. bry.* cocc. coloc. *cupr.* dig. *hyos.* lach. merc. nx-v. op. puls. stram. *verat.*
— see also under Delirium.

**Estranged** from her family: anac. con. hepar. nat-c. *nat-m.* phos. plat. *sep.*
— flies from her own children: lyc.

**Exaltation** of fancy: agar. agn. alum. ambr. am-c. anac. *ang.* ant-c. arn. ars. *asaf.* aur. *bell.* bry. calc. cann-i. canth. caus. *chin. coff.* con. croc. dig. graph. hyos. k-ca. *lach.* lact. lyc. meph. mur-ac. nx-v. olnd. *op.* oxal-ac. phos. ph-ac. puls. sabad. sil. stram. sulph. valer. verb. viol-od.
— See also Excitement.
— in company: pallad. sep.
— nervous: bell. coff. nx-v.
— then dull: anac.

**Excitability**: *acon.* arn. asar. carb-v. cham. chin. ign. marum. nit-ac. *nx-v. puls.* valer. verat.

**Excitement**: abrot. acet-ac. *acon.* agar. agn. alum. ambr. am-c. ang. ant-c. ant-t. arn. *asaf.* aur-m. bell. bry. calad. calc-p. camph. cann-i. cham. chel. chin. cic. clem. cob. cocc. *coff.* coloc. con. creos. croc. crotal. cubeb. cupr. cyc. dig. elaps. eucal. eup-per. ferr. fluor-ac. gels. glon. gran. graph. hell. hura. hydr-ac. hyos. iod. k-bi. k-ca. k-iod. lac-can. *lach.* laur. lachn. lil-t. lyc. mag-s. *marum.* meph. merc. merc-c. mill. mur-ac. nat-c. nat-m. nat-p. *nit-ac.* nx-j. *nx-v.* ol-jec. *op.* oxal-ac. petr. phos. ph-ac. *plat.* plect. psor. raph. rheum. rhus. sabad. sal-ac. samb. sang. secale. seneg. sep. *sil.* spong. *stram.* sulph. sul-ac. tabac. *tarent.* thea. thu. trill. valer. verb. viol-od. vip. znc. ziz.
— **alternating** with convulsions: stram.
— — — delirium: agar.
— **confusion**, as from: nx-m.
— **convulsive**: canth.
— **emotional**: alum. cann-i. phos.
— **feverish**: ant-t. chlor. colch. cubeb. merc. merc-c. phos. secale. seneg. sul.-i.
— — in evening: merc-c.
— — at night: sulph.
— — after dinner: sep.
— — during menses: rhod.

— **nervous**: ACON. arg-n. cinnb. phos. stryc. sul-ac. tarent. thea.
— **stomach**, with pain in pit of: mill.
— **tremulous**: aur.
— **vascular**: oxal-ac. sep.
— as from **wine**: lyc. mosch.
**Conditions** of excitement.
— **morning**: ars. æth. chin-s. cop. lach. *lyc.* mang. nat-m. sep.
— **afternoon**: aloe. ang. cann-i. iod. lyc.
— **evening**: am-c. carb-v. chel. daph. ferr. fluor-ac. lyc. mezer. nx-v. oxal-ac. phel. phos. sumb. znc.
— **night**: am-c. apis. dig. ferr. hura. nit-ac. plect. sep. sulph. thu.
— — during sleep: lyc.
— — on waking: cocc-c. thu.
— **beer**, after: cocc-c.
— **fever**, during: sulph. tarent.
— — with heat of head: meph.
— **menses**, before: mag-m.
— — during: cop. ferr. mag-m. rhod. *tarent.*
— — after: ferr.
— **micturition**, during: aloe.
— **music**, from: sumb. tarent.
— **sadness**, after: spig.
— **sleep**, after: sep.
— **talking**, when: merc.
— **tea**, after: sulph.
— **toothache**, after: sep.
— **walking**, after: caus. fl-ac. nat-m.
**Exclamation.** See Crying out.
**Expression.** See under Face.
**Extravagance**: am-c. bell. caus. chin. croc. iod. petr. phel. ph-ac. plat. stram. verat.
**Faces**, sees: ambr. apis. arg-n. calc. carb-an. caus. op. sulph.
— See also Faces, Images, etc., under Delirium and Delusions.
**Fall**, fancies objects around him would: hyos.
**Fancies**: agar. anac. ang. apoc. arn. bell. cann-i. chel. coca. coff. conv-d. elaps. hyos. lach. mag-m. meph. *merc.* op. petr. phos. plan. sabad. sep. sulph. verb. znc. ziz.
— See also Delusions.
— evening: am-c. anac. chel. sulph.
— night: ars. aur. *cham.* hipp. hydr. ign. petr. sep.
— after going to bed: hell. phos.
— on reading: mag-m.
— **confused**: hyos. phos. stram.
— **frightful**: hydr-ac. hydr. merc. stram.
— **lascivious**: am-c. anac. aur. calc. dig. *graph.* hipp. lyc. op. thu. verb.
— **vivid**: cyc. hell. hyos. *lach.* naja. stram.
— — when falling asleep: nat-m.
**Fatigue**, mental: ambr. asar. bar. *calc.* cham. cic. colch. graph. iod. laur. *led.* lyc. merc. nat-c. nat-m. nx-m. *nx-v.* petr. phos. pic-ac. *puls.* sars. selen. sep. spig. spong. staph. sulph. sul-ac. valer.
— after eating: lach.
— mental labor from brief: graph. ph-ac. pic-ac. staph.
— from reading: sil.
— — — and writing: cann-s. sil.
— from the pain: sars.
— after vexation: znc.
**Fault-finding.** See Censorious.
**Fear**: acet-ac. ACON. agn. aloe. alum. am-c. anac. ang. *ars.* asaf. ant-c. aur. bapt. bar. *bell.* borax. *bry.* calad. *calc.* camph. cann-s. carb-an. carb-v. caus. cham. chin. cic. cimic. coca. cocc. coff. coloc. con. crotal. cupr. daph. dig. dros. euph. gent. glon. graph. hell. *hepar.* hyos. hyper. ign. ipec. k-ca. k-iod. lach. lobel. lyc. mag-c. mag-m. manc. merc-i-r. mezer. *mosch.* murx. mur-ac. *nat-c.* nat-m. nicc. *nitr.* nit-ac. nx-v. *op.* petr. phos. *plat.* psor. *puls.* ran-b. raph. rheum. rhod. *rhus.* rhus-v. ruta. secale. sep. sil. spig. squil. staph. stram. sulph. sul-ac. tabac. tarent. tilia. valer. znc.
— compare throughout with Anxiety, and see Frightened.
— **accidents**, of: acon. gins.
— **animals**, of: caus. *chin.* hyos. stram.
— **alone**, of being: ars. calc. camph. con. dros. elaps. hyos. k-ca. lyc. ran-b. sep. stram.
— — lest he injure himself: ars.
— **apoplexy**, of: arg. fluor-ac. nat-c. phos.
— — at night, with feeling as if head would burst: aster-r.
— — during stool: verat.
— — on waking: glon.
— **approaching** him, of others: anac. *arn.* con. ign. iod. lyc.
— — in delirium: cupr.
— — children cannot bear to have any one come near them: *cina.*

— **bad** news, of hearing: dros. hydrph. nat-p.
— **bed**, of the: cann-s. canth. lyc. merc. nat-c.
— — to go to: *acon.*
— **bitten**, of being: hyos.
— **brain**, of softening of: abrot. asaf.
— **brilliant** objects, looking-glass, etc., fear of, or cannot endure: stram.
— **burden**, of becoming a: raph.
— **cholera**, of the: lach. nit-ac.
— **coal-scuttle** of, the: cann-i.
— **cold**, of taking: nat-c. sulph.
— — during the chill fears the heat; during heat fears the chill: sulph.
— **confusion**, that people would observe her: *calc.*
— **consumption**, of: calc. lac-can. paul-p. sep. tarent.
— — of lung being diseased: aral.
— **contagion** of: bov. calc.
— **corners**, to walk past pointed: arg-n.
— **crime**, as if he had committed a: nx-v.
— **crowd**, of going into a: ACON. aloe. am-m. ars. *aur.* bar. bufo. calc. cic. con. dios. ferr. graph. hepar. hydr-ac. k-bi. k-ca. led. lyc. nat-c. *nat-m.* phos. *puls.* rhus. selen. stann. tabac. tilia.
— — of public places: *arn.*
— **cutting** himself when shaving: calad.
— **danger**, of impending: ether. mac.
— — on going to sleep: coff.
— — See also Misfortune.
— **dark**, of the: *acon.* bapt. calc. lyc. *puls.* STRAM. valer.
— **dawn**, of the return of: k-iod.
— **death**, of: ACON. *agn.* aloe. alum. am-c. anac. *apis.* ant-cr. arn. *ars.* asaf. aur. bapt. bar. *bell.* bry. cact. calc. *cann-i.* canth. caps. caus. cimic. cocc. coff. cop. cupr. dig. gels. glon. graph. *hell.* *hepar.* hyos. ign. ipec. iris. k-ca. *lach.* led. lobel. lyc. mag-s. *mosch.* nat-m. nit-ac. *nitr.* nx-m. nx-v. op. oxal-ac. petr. *phos.* ph-ac. phyt. PLAT. podo. psor. puls. raph. rheum. rhus. secale. sep. stram. squil. tabac. tarent. tril. verat. verat-v. vinc. znc.
— — **alone**, when: arg-n. ars.
— — — — on going to bed: *ars.*
— — **heart** symptoms, during: asaf.
— — **predicts** the time: ACON.
— — **soon**, after awhile: *agn.*
— — **sudden**, of: *ars.*
— — **hot** stage during: calc. cocc. ipec. mosch. *nit-ac.* RUTA.
— — — **sweat**: nitr.
— — — **menses**: acon. plat. verat.
— — — **vomiting**: *ars.*
— **destination**, of being unable to reach his: lyc.
— **disaster**, of: elaps. lil-t. tabac.
— **disease**, of impending: ars. borax. calc. carb-an. cic. ether. hepar. hydr. iris. k-ca. lach. lil-t. nat-m. phos. thu. tril.
— — worse walking in open air: hepar.
— **dogs**, of: chin. stram.
— **downward** motion, dread of: borax.
— **drawn** upward, of being: camph.
— **drink**, of: plb.
— — during nausea: jatr.
— — during thirst: tarent.
— **driving** him from place to place: men.
— **drowned**, of being: cann-i.
— **eating**, of: caus. puls. tarent.
— — when hungry: grat.
— **everything** around, of: acet-ac. bell.
— **exertion**, of: calad. guai. mezer. phos. ph-ac. phyt. sul-iod. tabac. thea.
— **exposure**, night in bed, of: mag-c.
— **extravagance**, of: op.
— **eyes**, of closing, lest he might never awake: æth.
— **failure**, of: psor.
— **falling**, of: acon. alum. lac-can. lil-t. nx-v. stram.
— — evening: hydrph.
— — on turning head: derris.
— — when going to sleep: coff.
— — of letting things fall: coca.
— **fast**, dares not: creos.
— **fever**, while chilly: sulph.
— — while chilly on going to bed hura.
— — of typhus: tarent.
— **fit**, of having a: agar. arg. cann-i. nx-m.
— **fluids**, of: gels. hyos. nx-v.
— **food**, of: grat. op.
— **friends**, of: cedr.
— **future**, of the (about the): anac. ant-t. arn. bar. *bry.* calc. caus. chel. con. *dig.* dros. dulc. graph. k-ca. mang. nat-c. nat-m. puls. rhus-r. spig. spong. staph. stram. sulph.

— — weeping, amel.: dig.
— **gallows**, of the: bell.
— **ghosts**, of: acon. *ars.* bell. brom. cann-i. *carb-v.* cocc. dros. lyc. phos. puls. stram. *sulph.* znc.
— — at night: ars. carb-v. chin. *puls.* ran-b. *sulph.*
— **grieving**, as if: phos.
— **happen**, as if something would: cact. elaps. hydrph. nat-ars. phos. pyrus.
— — when alone, relieved by conversation: ratan.
— **heart** disease, of: lac-can. lach.
— — fears heart will cease to beat unless constantly on the move: gels.
— **health**, that she has ruined: chel.
— **hurt**, of being: calad. cann-i. stram.
— **imaginary** things: bell. laur. merc.
— **imbecile**: stram.
— **insanity**, of: acon. *alum.* ambr. arg-n. CALC. CANN-I. carb-an. chel. chlor. cimic. cupr. graph. iod. k-bi. lac-can. lil-t. merc. nat-m. nx-v. phos. phys. plat. stram. tarent. thu.
— — at night: phys.
— **jealousy** and tearfulness with fear of death: apis.
— **joints** are weak, that: sep.
— **jumps** out of bed from fear: ars.
— — on touch: bell.
— **killing**, of: absin. *rhus.*
— — with a knife: ars. derris.
— **lung** is diseased, that: aral.
— **medicine**, of taking too much: all-s. iber.
— **men**, of: bar-ac. puls.
— — See also Persons.
— **mirrors** in room, of: camph. stram.
— **mischief**, he might do, night on waking: phys.
— **misfortune**, of: acon. agar. alcoh. alum. anac. ant-c. arn. ast. atrop. calad. cact. calc. *chin-s.* cic. clem. colch. cyc. dig. dros. ferr. fluor-ac. gins. glon. graph. hydr-ac. hydrph. iod. k-iod. lach. lil-t. mag-c. mag-s. men. merc-c. mezer. nat-p. nat-s. nicc. nx-v. psor. rhus. rumx. stram. sulph. tabac. valer. vinc.
— — morning: am-c. mag-s.
— — afternoon: cast. hura.
— — evening: ferr. nat-m.
— — evening in bed, better: mag-c.
— — during chilliness: cyc.
— — — fever: atrop.
— **murdered** of being: op. phos. stram.
— — of committing murder: ars. sulph.
— **noise**, of sudden: *cocc.*
— **objects**, of bright: cann-i.
— **observed**, her condition, of being: *calc.*
— **occupation**, of: selen:
— **others**, for: sulph.
— **pain**, of: cori-r. derris. pip-m.
— — See Suffering, Fear of.
— **paralysis**, of: asaf.
— **persons**, of: acon. aloe. am-m. ars. *aur.* bar. bar-ac. calc. cic. *con.* croton. cupr. dios. ferr. graph. hepar. k-bi. k-ca. *led.* lyc. nat-c. *nat-m.* phos. *puls.* selen. sep. stann. tabac. tilia.
— **physician**, will not see her, he seems to terrify her: iod. verat-v.
— **pneumonia**, of: chel.
— **pointed** things, of: spig.
— — — in dreams: merc.
— — — in delirium: sil.
— **poisoned**, of being: all-s. *bell.* bry. glon. *hyos.* lach. rhus. verat-v.
— — has been poisoned: glon.
— **public** places, of: *arn.*
— — See also Crowd, fear of, etc.
— **putrify**, body will: bell.
— **rain**, of: elaps.
— **reason**, of losing. See Insanity, Fear of.
— **recover**, will not: all-s. ant-t. calc. glon. k-ca. tarax.
— **respiration**: osm.
— **rise**, fears to, as if exhausted after a long walk, better after rising: nx-v.
— **robbers**, of: alum. ars. aur. bell. con. elaps. *ign.* lach. mag-c. merc. nat-c. *nat-m.* phos. sil. sulph. verat. znc.
— — midnight on waking: ign. sulph.
— — Compare with Thieves under Delusions.
— **ruin**, of financial: ambr. *calc-fl.* *sep.*
— **run** over on going out: anthr. phos.
— **sad**: *dig.* *nat-m.*
— — worse from music: *dig.*
— **salvation**, of his: cann-i. verat.
— — See also under Despair, Anxiety, etc.
— **say** something wrong, fear lest he should: lil-t.
— **sleep**, to go to: led. merc. nx-m.
— — fears to close the eyes lest he never wake: æth.
— — he will never sleep again: *ign.*

— **sobbing** during sleep: ipec.
— **society**, of: hell. tilia.
— — of his position in: verat.
— **sold**, of being: hyos.
— **solitude**, of: *ars.* asaf. bell. bism. cadm. camph. clem. con. dros. elaps. gal-ac. hyos. k-ca. *lyc.* plb. sep. stram. tabac. verat.
— — evening: ran-b. tabac.
— **spectres**, of. See Ghosts.
— **stomach**, of ulcer in: ign.
— **strangers**, of: ambr. bar-ac. caus.
— **suffering**, of: bry. calc. eup-per. lil-t.
— **suffocation**, of: acon. carb-an. ether. merc. stram.
— — night: agar.
— — fears to go to sleep for fear of: bapt.
— **superstitious**: rhus.
— **thunder**, of: nat-m.
— **touch**, of: *arn.* coff.
— — See Touch in general.
— **tread** lightly, must, or will injure himself: cupr.
— **tremulous**: *aur.* ratan.
— **trifles**, of: *ign.*
— **troubles**, of imaginary: hydr-ac.
— **unaccountable**: alcoh.
— **vertigo**, of: sumb.
— **voice**, of using: cann-i.
— **walking**, of: nat-m.
— — across busy streets: *acon.*
— **water**, of: cann-i. *hyos.* phel. stram.
— **wet** his bed, fears he will: alum.
— **wind**, of: *cham.*
— **women**, of: raph.
— **work**, of: *arg-n.* calc. cham. hyos. petr. *puls.* ran-b. *sil.* *sulph.* tabac. tarax. tong.
— — during headache: gran.
— — of literary: nx-v. *sil.* sulph.

**Conditions** of Fear.
— **morning**: arg-n. caus. graph. mag-s. nicc. sul-ac.
— **afternoon**: æth. berb. carb-an. cast. nicc. stront. tabac.
— **evening**: alum. bar. brom. calad. calc. carb-an. caus. cocc-c. dros. form. hipp. k-ca. lach. lyc. mag-m. merc. nat-c. nat-m. nx-v. *phos.* *puls.* ran-b. rhus. stront. tabac. valer. verat.
— at **night**: am-c. *ars.* *bell.* carb-an. carb-v. caus. *chin.* cocc. con. dros. graph. hepar. ipec. lach. lyc. merc. nat-c. nat-m. nit-ac. phos. *puls.* *rhus.* sil. stram. *sulph.* znc.
— — amel. in bed, at: mag-c.
— — after waking: con.
— **air**, in open, amel.: plat.
— **alone**, when: ars. brom. con. dros. k-ca. lyc. ran-b.
— **approach**, at the, of others: acet-ac. *arn.* cadm-s. cann-i. cupr. iod. op.
— — — lest he be touched: ARN.
— **crowd**. See Crowd, fear of going into a.
— **dark**, in the: lyc. stram. valer.
— **dinner**, after: mag-m. phel.
— **emission**, after an: aloe. carb-an.
— **food**, after: canth. caus. mag-m. phel. tabac.
— **headache**, during: glon.
— **heart**,, from pain at: daph.
— **house**, on entering: plat. tilia.
— **labor**, after: iod.
— **looking**, before her, when: sulph.
— **menses**, before: *acon.* calc. k-bi. plat. secale. sulph. xanth.
— — during: acon. mag-c. phos. plat. secale.
— — during menstrual colic: ant-t.
— **music**, from: dig.
— **nausea**, after: tabac.
— **near**, of those standing: bell.
— **noise**, from: aloe. alum. cann-s. caus. chel. *cocc.* hipp. hura. mosch. nat-c. sabad. tabac.
— **pregnancy**, during: hydrph.
— **respiration**, during painful: viol-od.
— **riding**, when: lach. sep.
— **room**, in: alum.
— **sitting**, when amel.; iod.
— **sleep**, before going to: calad. rhus.
— — during: merc-i-r.
— **supper**, after: caus.
— **thinking** of disagreeable things, when: phos.
— — — of sad things: rhus.
— **throat**, from sensation of swelling of: *glon.*
— **waking**, on: bell. carb-an. lept. nat-p. nx-v. puls. tereb.
— — from a dream: bov. ph-ac. sil.
— **walking**, while: alum. bar-ac. cina. *lyc.*
— **weeping** amel.: dig. graph. tabac.

**Fearfulness**. See Anxiety, Fear, etc.
**Fickle**. See Inconstant, Irresolute, etc.
**Fidgety**. See Restlessness.
**Fire**, wants to set things on: hepar.
— a flame of, seemed passing through him: phos.

